# Understanding the role of welfare state characteristics for health and inequalities – an analytical review

**DOI:** 10.1186/1471-2458-13-1234

**Published:** 2013-12-27

**Authors:** Kersti Bergqvist, Monica Åberg Yngwe, Olle Lundberg

**Affiliations:** 1 Centre for Health Equity Studies, Stockholm University/Karolinska Institutet, Stockholm, Sweden; 2 Department of Health Sciences, Mid Sweden University, Östersund, Sweden

**Keywords:** Health inequalities, Health, Welfare regime, Social expenditure, Welfare institutions

## Abstract

**Background:**

The past decade has witnessed a growing body of research on welfare state characteristics and health inequalities but the picture is, despite this, inconsistent. We aim to review this research by focusing on theoretical and methodological differences between studies that at least in part may lead to these mixed findings.

**Methods:**

Three reviews and relevant bibliographies were manually explored in order to find studies for the review. Related articles were searched for in PubMed, Web of Science and Google Scholar. Database searches were done in PubMed and Web of Science. The search period was restricted to 2005-01-01 to 2013-02-28. Fifty-four studies met the inclusion criteria.

**Results:**

Three main approaches to comparative welfare state research are identified; the Regime approach, the Institutional approach, and the Expenditure approach. The Regime approach is the most common and regardless of the empirical regime theory employed and the amendments made to these, results are diverse and contradictory. When stratifying studies according to other features, not much added clarity is achieved. The Institutional approach shows more consistent results; generous policies and benefits seem to be associated with health in a positive way for all people in a population, not only those who are directly affected or targeted. The Expenditure approach finds that social and health spending is associated with increased levels of health and smaller health inequalities in one way or another but the studies are few in numbers making it somewhat difficult to get coherent results.

**Conclusions:**

Based on earlier reviews and our results we suggest that future research should focus less on welfare regimes and health inequalities and more on a multitude of different types of studies, including larger analyses of social spending and social rights in various policy areas and how these are linked to health in different social strata. But, we also need more detailed evaluation of specific programmes or interventions, as well as more qualitative analyses of the experiences of different types of policies among the people and families that need to draw on the collective resources.

## Background

In the area of health inequality research, as well as in the wider fields of social and public health sciences, there is an on-going and ever developing discussion on *macro* versus *micro* level explanations. The terminology used varies, but the core issue remains the same; what are the most important factors behind poor health and health inequalities, the upstream or downstream, distal or proximal, structural or individual ones? From an etiological or causal point of view, this duality can at least in part be resolved by building more complex models, where different levels of mechanisms are nested and organised sequentially. From a policy point of view, this sort of understanding is also important, but there is still the issue of where to find the best policy entry points.

Here the recent years’ work on social determinants have constituted an attempt to draw the attention of policy makers to the *causes of the causes*, in other words to the wider social circumstances in which people live their lives and that more or less indirectly affect their chances to be healthy and live long. The renewed interest in public health for more general conditions of life such as childhood and conditions of upbringing, education and training, work and economic resources has also led to a larger focus on the wider policy context. If we think that the causes of the causes are embedded in conditions of life more generally, then we also need to look for policy options wore widely. More concretely, this means that a broad range of policies and programmes dealing with and affecting education, work and incomes of people becomes of great interest also from a public health perspective.

These sets of policies are in turn often not combined randomly. On the contrary, a long and rich research tradition has studied how different welfare states vary systematically in the principles that have guided the design and execution of policies for poverty alleviation, to take but one example. In this tradition of ‘Welfare State Research' , one prominent question has been the driving force behind the growth of modern welfare states, while another key issue has been the consequences of different types of welfare state solutions in terms of e.g. poverty or fertility rates. With a growing interest in public health research for the wider policy context, it is quite natural that the past decade has witnessed a growing body of research on welfare states characteristics and health inequalities. The logic behind this is undisputable – if it can be argued that some types of welfare states are more successful in combating poverty and deliver a decent life to a larger share of the population there is reason to believe that improvements in the level and distribution of the ‘causes of the causes’ will also lead to better health and smaller inequalities.

However, while early comparative studies suggested that countries like Sweden had smaller inequalities than countries like Great Britain [1], larger and more systematic comparative studies have not been able to demonstrate clear differences in health inequalities that match traditional welfare state clusters [2]. In addition, some recent reviews of the field have found clearly mixed results [3,4].

The mixed findings provide a challenge for welfare state research and public health research alike, and even more so for policy making. If social determinants and ‘causes of the causes’ can be demonstrated to be important, why is it so hard to get consistent results when analysing welfare states and health inequalities? One possible answer to this important question may be that there are substantial theoretical and methodological differences between studies that at least in part lead to different findings. A fundamental issue is that there are several ways of analysing welfare states and health inequalities in comparative health research.

Dahl and van der Wel [5] describe three common approaches to characterise the welfare state; as regime types, as welfare institutions or as social spending. Comparative health research has been dominated by the ‘*Regime approach' *, in which classifications of countries based on various political elements are used. Those who support this approach have argued that certain countries cluster together in ‘welfare state regimes’ based on similar ideologies and policies or political traditions. One cluster of countries may, for example, support universal access to different services while another operates on the individual’s private responsibility to take care of and handle one’s own welfare, and that only the most poor qualify for social support. The general idea is that by specifying ideal types it is possible to assess the underlying commonalities and principles of social structures and welfare institutions [5]. In addition, the Regime approach comes in a variety of versions that differ both in terms of theoretical and empirical foundations and the countries included. By design, these ideal types will not fit the complex reality perfectly and might therefore give a rather crude result. Hence, this approach might be less useful in capturing mechanisms that generate inequalities in health. To complement the picture it may be important to also look at characteristics of social, health and labour market policy [5].

Another common approach is the ‘*Institutional approach*' , which focuses more on how welfare institutions and specific social policies and programmes are designed and how these translate into population health. The Institutional approach addresses the characteristics of policy programmes for, for example, pensions, sickness pay, unemployment benefit, family policies and work accidents. These characteristics may for example be qualifying criteria, replacement rates, duration and coverage [6]. Several international comparative databases (e.g. the Social Citizenship Indicator Programme, SCIP) provide historical information on such characteristics including policy programmes. In order to construct relevant programme features the databases apply a number of assumptions regarding for example, age and family situation of a ‘standard worker’ [6]. This could be problematic if there are important groups that fall outside the living situations captured by these type cases.

A third approach is the ‘*Expenditure approach*' , which focuses on welfare state effort and generosity by concentrating on public spending on social protection and services. The spending on social protection and services is often expressed in terms of percentage of the Gross domestic product (GDP). The rationale for this is that ‘the government should be transferring relatively the same level of social expenditure as other nations in order to be considered as providing an equivalent degree of generosity and protection’ [7]. The spending approach has been criticised for its inability to differentiate between effort and need – a large spending on unemployment benefits and programmes may simply reflect a larger share of unemployed and not a higher ambition in terms of coverage or replacement rates [8]. Recent studies have tried to overcome this problem by weighting procedures [5], and their analyses also suggest that different choices regarding the spending variable (gross/net, absolute/relative) have little impact on the results.

We are not the first to point out that there are complications with these approaches (e.g. [5]), but we believe there to be a need to clarify and describe why results are as diverse as research suggests. To our knowledge, we are the first to stratify these studies according to how they are classified in each approach, for example by welfare regime typology, something that will complement existing research.

If we look at the existing literature with a more analytical view, taking into account methodological and theoretical differences, we might be able to sort out substantial findings from ‘noise’ caused by methodological and other shortcomings. Therefore, we aim to review the literature on welfare state, health and health inequalities, taking earlier reviews as our starting point. Our analytical approach is to further classify these later studies, published 2005 and later, according to their principal way to characterise the welfare state; as regime types, as welfare institutions or as social spending. Since the regime type approach is dominating, we also attempt at further distinctions within this category in order to find patterns that might explain the inconsistent results. Based on this analytical framework, we discuss the general findings in the literature, comment on the different investigative approaches, and point to where substantial conclusions about welfare state policies and health inequalities can be made.

## Methods

### Search strategy

**Figure 1:**
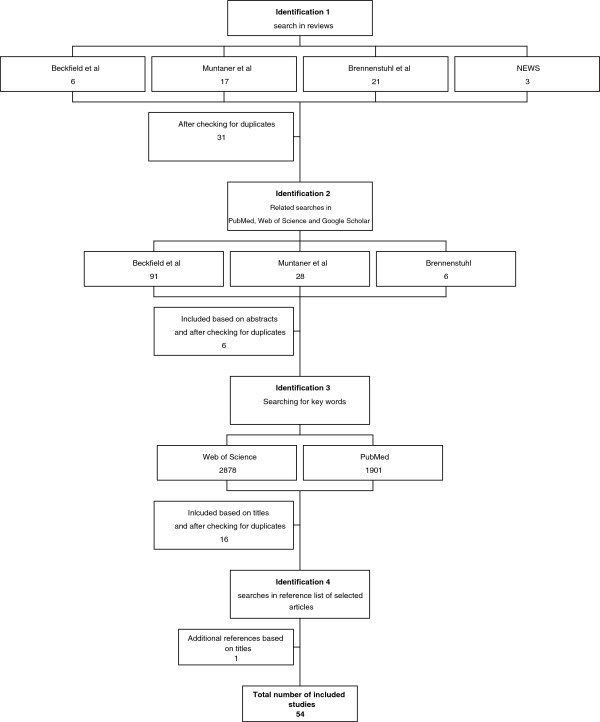
**Summary of search strategy.** Summary of the search strategy used; the different identification methods and study selections.

This review adopted several search strategies to detect relevant studies [see Figure 1]. The first step was to manually explore three large reviews related to the subject; by Beckfield and Krieger [9], Muntaner et al. [4] and Brennenstuhl et al. [3]. The NEWS report [10] was also explored since it includes studies relevant to the Institutional approach. Studies related to any of the three core approaches were selected, studies with themes such as globalisation or democracy were excluded. After reading the abstracts of the related studies, six studies were selected from Beckfield and Krieger [9], 17 from Muntaner et al. [4], 21 from Brennenstuhl et al. [3] and three relevant studies were selected from the NEWS report. This strategy yielded 31 exclusive studies. The selected articles were thoroughly read in order to make sure they were placed in the correct “pile of approaches”.

The second step was to search for ‘related articles’ to the three reviews in PubMed, Web of Science and Google Scholar. In order to get the most recent publications the strategy was to find articles that have cited any of the three reviews. Brennenstuhl et al. [3] (published in 2012) had not been cited any time in Web of Science, once in PubMed and five times in Google Scholar (2 articles selected). Muntaner et al. [4] (published in 2011) was cited eight times in Web of Science, twice in PubMed and 18 times in Google Scholar (1 article was selected). Finally, Beckfield and Krieger [9] (published in 2009) was cited 31 times in Web of Science (4 articles selected), seven times in PubMed (1 article selected) and 53 times in Google Scholar (4 articles selected). This strategy yielded six related exclusive studies [5,11-15].

The third step was to search for key words in Web of Science and PubMed. The searches were limited to the period 20100101–20130228 and was based on the fact that the reviews were published in 2009 [9], 2011 [4] and 2012 [3] and would therefore hopefully exhaust the number of articles prior to 2010 in this field of research. Searches were based on search terms related to the approaches mentioned above leading to three sets of search strings each combining the related terms with the health terms. Attention was put on health inequalities. An initial screening was done by looking at the titles, and thereafter the abstracts of relevant articles were read.

The first search string combined welfare regime related terms and health inequalities ((welfare state* or welfare typ* or welfare regime*) AND (health inequalit* or health inequit* or health disparit*) AND (Humans[Mesh])) and resulted in 182 citations in Web of Science and 622 citations in PubMed. Seven relevant “new” studies were found.

The second search string combined policy-related terms and health inequalities ((social polic* or health polic* or family polic* or pension polic*) AND (health inequalit* or health inequit* or health disparit)* AND (Humans[Mesh])) and resulted in 2230 citations in Web of Science and 654 citations in PubMed. Two relevant “new” studies were found.

The third search string combined expenditure-related terms and health inequalities ((spending or expenditure or welfare state generosity) AND (health inequalit* or health inequit* or health disparit*) AND (Humans[Mesh])) and resulted in 466 citations in Web of Science and 625 citations in PubMed. Seven relevant “new” studies were found.

The fourth, and final, step was to manually explore the reference lists of the selected articles. This resulted in one study [16], relevant to the ‘Institutional approach’.

### Inclusion criteria

Articles selected for this review had to be an empirical peer-reviewed study published in English in an International journal. They had to be published between January 2005 and February 2013 in order to get recent results. Studies should address any type of health outcome (both morbidity and mortality measures) and preferably social inequalities in health (stratified by education, income or other relevant measure). Studies examining health inequalities based on ethnicity and minority groups were excluded. The study population could be from all age groups. Studies using data from industrial countries including East Asia were included.

Specific inclusion criteria for the ‘Regime approach’ were that studies should include cross-national comparisons of different health outcomes. The countries in the analysis could either be groups of countries or typical representatives of a welfare regime, or other cross-national country or regional comparisons. The number of countries used for comparisons had to be at least two countries.

A specific inclusion criterion for the ‘Institutional approach’ was that focus should be placed on welfare state indicators (replacement rates and coverage of specific social policies such as pensions and family benefits) and levels of generosity in social policy delivery, and look at to what extent variations in generosity and/or coverage are linked to variations in different health outcomes.

A specific inclusion criterion for the ‘Expenditure approach’ was that studies should examine different levels of social/health spending or social transfers measured as government health or social spending.

## Results

**Figure 2:**
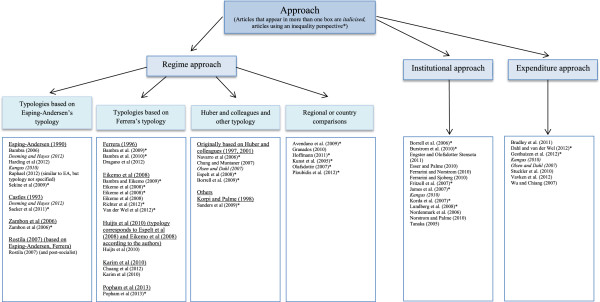
**The three approaches used in comparative welfare research.** Figure illustrating the three main approaches to comparative research as well as the authors of the studies included in each approach. The Regime approach is further divided based on main typology used.

The total number of studies selected for this review is 54 [see Figure 2]. Thirty-four studies have been selected for the ‘Regime approach' , 14 studies for the ‘Institutional approach’ and eight for the ‘Expenditure approach’. One study [17] has been placed in all three approaches.

The studies in the ‘Regime approach’ have been sorted according to different criteria; by outcome (both by morbidity and mortality outcomes, but also according to where health was found to be the best), by type of data used, and by the number of countries under study. Since no clear results have been found we have decided to analyse and summarise the characteristics and findings of the different groups of typology/country comparisons. A short section of the other results can be found after the Regime approach results.

The studies in the ‘Institutional approach’ have been sorted according to policy theme and the studies in the ‘Expenditure approach’ have been sorted by either social or public spending.

### The Regime approach

The ‘Regime approach’ is the largest approach with 34 studies fitting the criteria. An Additional file shows the descriptive characteristics of the studies [see Additional file 1]. This approach is the most common way of examining cross-national welfare state comparisons of health and health inequalities and there seems to be a pattern of increased popularity with time. Detailed timelines of the studies’ publication year can be found in an Additional file [see Additional file 2]. Among the 34 studies in the ‘Regime approach' , 26 examined overall population health and almost two thirds of the studies (n = 21) examined socioeconomic inequalities in health (some studies look at both). More than a third of these (8/21) examine inequalities by social class, which is often based on two or more variables related to education and income. Different variables are used to measure health; self-rated health is the most common measure (13/34). When health measures are categorised as either mortality or morbidity related measure one finds that it is more common to use morbidity measures (32 compared to 18). Most studies use a typology approach (28), and those based on Ferrera’s typology [18] are most common.

### Different typologies

The three main regime typologies dominating the ‘Regime approach’ are the typologies by Esping-Andersen [19], Ferrera [18] and Huber and colleagues [20,21] [see Table 1]. This has also been found in the review by Brennenstuhl et al. [3].

**Table 1:** The three main typologies used in the ‘Regime approach’

**Author**	**Main elements**	**Welfare regime typologies**			
Esping-Andersen [19]	- Decommodification	**Liberal**	**Conservative**	**Social democratic**	
	- Social stratification	Australia	Finland	Austria	
	- Private-public mix	Canada	France	Belgium	
		Ireland	Germany	Denmark	
		New Zealand	Italy	The Netherlands	
		UK	Japan	Norway	
		USA	Switzerland	Sweden	
Ferrera [18]	- Coverage	**Anglo-Saxon**	**Bismarckian**	**Scandinavian**	**Southern**
	- Replacement rates	Ireland	Austria	Denmark	Italy
	- Poverty rates	UK	Belgium	Finland	Greece
			France	Norway	Portugal
			Germany	Sweden	Spain
			Luxembourg		
			The Netherlands		
			Switzerland		
Huber and colleagues [20,21]	- Prevailing political tradition	**Liberal**	**Christian democratic**	**Social democratic**	**Wage-earners**
		Canada	Austria	Denmark	Australia
		Ireland	Belgium	Finland	New Zealand
		UK	Germany	Norway	
		US	France	Sweden	
			Italy		
			The Netherlands		
			Switzerland		

These three typologies have in the studies selected for this review often been modified by either adding a regime type or adding countries to existing regime types, thereby changing the set-up of the typology somewhat. For a full table of typologies used in the studies selected see an Additional file [Additional file 3]. The most influential typology by Esping-Andersen [19] is constituted by three regime types in which highly developed countries (mainly European) were fitted [see subsection ‘Theoretical description- Esping-Andersen’ for further information]. With time, additional regime types have been added by various authors. The typology by Ferrera is often described as being based on the work by Esping-Andersen but includes features that makes it stand on its own [see subsection ‘Theoretical description- Ferrera’]. Quite a few authors have chosen to base their modification on Ferrera’s typology rather than Esping-Andersen’s. For example, Eikemo et al. e.g. [22] add a fifth regime type, the Eastern European, and Karim et al. [23] add a sixth, the East Asian. The typology by Huber and colleagues [20,21] has also been modified by many [see subsection ‘Theoretical description- Huber and Stephens’]. Navarro for example, together with different co-authors, have modified it in different articles by removing one regime type and adding a new one [24-26].

Most countries, regardless of the typology used, remain in some sort of core regime cluster. Austria and The Netherlands sometimes move from the Conservative/Bismarckian/Christian democratic group to the Social democratic group but the majority of research tends to label these countries as Conservative. Australia sometimes moves from the Liberal/Anglo-Saxon cluster to a separate group called Radical/Targeted/Wage-earner.

### Esping-Andersen

The original Esping-Andersen typology with three welfare state clusters is used by four out of nine studies [17,27-29]. The remaining five studies have used modified versions by adding a Radical regime [30,31], a Latin regime [32], or a Mediterranean and Eastern/Post-communist/Post-socialist regime [33,34]. Self-rated health and life expectancy are the most common health measures and are used in three studies respectively. Mortality measures are almost as common as morbidity measures. Descriptive characteristics of studies in the Esping-Andersen group are shown in Table 2.

**Table 2:** Descriptives of the Esping-Andersen group

**Descriptive measure**	**N (author)**
**Data used**	
- OECD, WHO, UN	3 (Bambra, Kangas Zambon*)
- Longitudinal data	3 (Harding*, Sekine*, Sacker*)
- ESS	1 (Rostila)
- Human mortality database	1 (Kangas)
- World values survey	1 (Deeming & Hayes)
**Health outcome**	
- Self-rated health	3 (Sacker*, Zambon*, Rostila)
- Life expectancy	3 (Kangas, Raphael, Rostila)
- Infant mortality	2 (Bambra, Raphael)
- Mortality	1 (Harding*)
- Change in life expectancy at birth	1 (Kangas)
- Health symptom load	1 (Zambon*)
- General wellbeing	1 (Zambon*)
- Health behaviours	1 (Zambon*)
- Self-reported happiness	1 (Deeming & Hayes)
- The Short-Form 36:	
○ Physical health	1 (Sekine*)
○ Mental health.	1 (Sekine*)
**Health outcome**	
- Morbidity	9
- Mortality	7
**Measures inequality (total)**	4 (Harding*, Sekine*, Sacker*, Zambon*)
- SEP (social class/SES)	3 (Sekine*, Sacker*, Zambon*)
- Employment status	1 (Harding*)

Many articles examined multiple outcomes and hence the number of studies using the different health outcomes (16) is greater than the number of studies (n = 9).

Mortality measures include: life expectancy, infant mortality, mortality rate, change in life expectancy at birth.

Morbidity measures include: self-rated health, self-reported happiness, health symptom load, general well-being, health behaviours, physical and mental health functioning.

* = Studies with an inequalities perspective.

Seven studies examine population health. Four of these find that health is better in the Nordic countries. The measures of health are infant mortality [27,32] self-reported happiness [30] and mortality rate [28] (for women). One study finds that male mortality rates are better in Conservative regimes [28]. Other studies find that health is better in other regimes (mainly Southern and Central European regimes), regarding outcomes such as life expectancy [17,34] and self-rated health [29,34]. Four out of nine studies examine inequalities in health and results regarding differences between regimes vary. One study finds some evidence of smaller inequalities in Social democratic countries for men but not for women [29]. The three other studies find that health inequalities in mortality [28] and self-rated health [31] are smaller in Conservative regimes. Another finds that inequalities in self-rated health are smaller in Liberal and Eastern regimes, and inequalities in general wellbeing and health symptom load are smallest in Liberal and Southern regimes [33]. For further information on study characteristics and results see an Additional file [see Additional file 4: Esping-Andersen].

### Theoretical description- Esping-Andersen

Esping-Andersen’s (E-A) typology proposed in ‘The three worlds of welfare capitalism’ [19] in 1990 is the most well-known and has been criticised and modified by many (e.g. [18,35]). Eighteen Organisation of Economic Cooperation and Development (OECD) countries were categorised into ideal clusters of welfare states based on principles regarding unemployment, sickness and pension benefits [36]. The clusters of countries are classified according to three principles:

– Decommodification: the extent to which an individual’s welfare is reliant upon the market, particularly in terms of pensions, unemployment benefit and sickness insurance

– Social stratification: the role of welfare states in maintaining or breaking down social stratification; and the

– Private-public mix: the relative roles of the state, the family, the voluntary sector and the market in welfare provision.

The operationalisation of these principles was based mainly on decommodification indexes and led to three ideal welfare regime types [19,36]:

– *Liberal* countries where policies are based on the idea that people are responsible for their own welfare. In other words, state welfare provision is minimal, benefits are modest and the criteria for entitlement are often strict, and recipients are usually means-tested and stigmatised. The countries in the liberal regime type are Australia, Canada, Ireland, New Zealand, UK and the USA.

– *Conservative* countries where access to social support is often earnings-related and administered through the employer. It is distinguished by its “status differentiating” welfare programmes which tend to maintain existing social patterns. These regimes are usually shaped by historical church traditions and the role of the family is emphasised. The countries in the conservative regime type are Finland, France, Germany, Italy, Japan and Switzerland.

– *Social Democratic* countries which belong to the smallest regime cluster and are quite the opposite of liberal and conservative welfare regimes in that there is a public responsibility for welfare and that access to services and benefits is universal. Welfare provision is characterised by comparatively generous benefits, a commitment to full employment and income protection, and a strongly interventionist state used to promote equality through a redistributive social security system. The countries in the social democratic regime type are Austria, Belgium, Denmark, the Netherlands, Norway and Sweden.

This typology has offered an influential typology that has encouraged much research. E-A’s main goal was to describe relationships between states, labour markets, and families and his typology is based on characteristics that refer to both institutions and outcomes [37]. It is important to remember that it was not developed to account for cross-national differences in health or health inequalities [5] and one must therefore bear in mind that it does not necessarily mean that this typology should have an immediate and direct effect on health and health inequalities.

#### Modified version

Castles and Mitchell

Castles and Mitchell [35] have, in 1993, built on E-A’s typology and focus on welfare state differences in redistribution of social transfers and provision of welfare services [31]. Different countries levels of aggregate expenditure and degree of benefit equality were examined [36] and based on this analysis they argued that Australia, New Zealand and the UK made up a targeted welfare state, the Radical welfare state.

### Ferrera

The original Ferrera typology is used by three out of 13 studies [36,38,39]. The remaining 10 have all modified the typology by adding Eastern Europe, and in some cases East Asia. Morbidity measures are more common to use. Self-rated health is the most common and is used in eight studies. Descriptive characteristics of studies in the Ferrera group can be found in Table 3.

**Table 3:** Descriptives of the Ferrera group

**Descriptive measure**	**N (author)**
**Data used**	
- ESS	6 (Bambra et al.*, Bambra & Eikemo*, Eikemo et al.*, Eikemo et al.*, Eikemo et al., Huijts)
- OECD, WHO, UN	3 (Richter*, Chuang et al., Karim et al.)
- SHARE	1 (Dragano)
- Human mortality database	1 (Popham*)
- EUROTHINE	1 (Bambra et al.*)
- Longitudinal data	1 (Dragano)
- EU-SILC	1 (Van der Wel*)
**Health outcome**	
- Self-rated health	8 (Bambra et al.*, Bambra et al.*, Bambra & Eikemo*, Eikemo et al.*, Eikemo et al.*, Eikemo et al., Richter*, Huijts)
- Longstanding limiting illness	5 (Bambra et al.*, Bambra & Eikemo*, Eikemo et al.*, Eikemo et al.*, van der Wel*)
- Infant mortality	3 (Chuang et al., Karim et al., Popham*)
- Life expectancy	3 (Chuang et al., Karim et al., Popham*)
- Depressive symptoms/depression	1 (Dragano)
- Psychosocial quality of work	1 (Dragano)
- Health complaints	1 (Richter*)
**Health outcome**	
- Morbidity	16
- Mortality	6
**Measures inequality (total)**	8 (Bambra et al.*, Bambra et al.*, Bambra & Eikemo*, Eikemo et al.*, Eikemo et al.*, Richter*, van der Wel*, Popham*)
- Education	4 (Van der Wel*, Eikemo*, Bambra et al.*, Bambra et al.*)
- SEP (social class)	1 (Richter*)
- Income	1 (Eikemo*)
- Employment status	1 (Bambra & Eikemo*)
- Popham measure (total inequality)	1 (Popham*)

Many articles examined multiple outcomes and hence the number of studies using the different health outcomes (22) is greater than the number of studies (n = 13).

Mortality measures include: life expectancy, infant mortality.

Morbidity measures include: self-rated health, limiting longstanding illness/morbidity/disability, depression/depressive symptoms, psychosocial quality of work, health complaints.

* = Studies with an inequalities perspective.

Nine studies report results related to population health. Two studies find that health (limiting longstanding illness, psychosocial quality of work and depressive symptoms) is better in Scandinavian countries compared to other regimes [38,40]. One study finds that health complaints are lower in Scandinavian regime and higher in Eastern and Southern regimes [12]. Two studies look at both infant mortality and life expectancy and both find that infant mortality rates are lowest in Scandinavian countries and that life expectancy is highest in East Asian countries [23,41]. Another study finds that life expectancy is higher in the Nordic countries for men, but for women it is higher in Confucian countries (East Asian) [11]. The same authors find that younger age mortality is lower in the Nordic countries but not for older age mortality. Four studies find that health is not the best in the Nordic countries. They instead find that self-rated health is better in Anglo-Saxon countries [22,42], in Bismarckian countries [43] or in Southern countries [12]. Limiting longstanding illness seems to be least reported in the Southern regimes [22,43].

Eight out of 13 studies look at inequalities in health and results regarding differences between regimes vary. Some find support of the Nordic countries performing better and having smaller inequalities while others find the opposite. Results vary by health outcome and gender making it difficult to draw any clear-cut conclusions. One study finds differences by welfare state regime, with inequalities being largest in Anglo-Saxon, Bismarckian and Scandinavian regimes [44]. They also find that women seem to be more affected by unemployment in the Scandinavian countries. Others find that health inequalities by income [22] and education [43] generally seem to be the smallest in Bismarckian regimes. Another study finds that inequalities in life expectancy are the smallest in the Southern regimes for women and are for men smaller in the Nordic countries, but the inequalities are measured as individual variation mainly [11]. Another study finds that social inequalities in sickness were lowest in the Southern regime for men and for women inequalities were lowest in Scandinavian regimes [40]. Drawing general conclusions are further complicated by results that vary by cohort and gender [36]. Some studies find no differences [12] or no consistent welfare regime patterning [39]. For further information on study characteristics and results see an Additional file [see Additional file 4: Ferrera].

### Theoretical description- Ferrera

After an extensive debate about E-A’s typology, Ferrera [18] introduced a modified typology in 1996 by focusing more on differences in how the social benefits are delivered as compared to E-A’s, where quantity of welfare provided was emphasised [45]. In doing this a new regime type, the Southern, was included. This lead to a typology with four different regime types; the Scandinavian (Social democratic), the Bismarckian (Conservative), the Anglo-Saxon (Liberal) and the Southern European (Italy, Greece, Portugal and Spain). Southern welfare states are described as ‘rudimentary’ [18] because they are still characterised by a highly fragmented system of welfare provision and welfare services [46]. Another prominent feature is the reliance on the family and voluntary sector [18,36].

#### Modified versions

Eikemo and colleagues

Another advancement in the welfare regime research has been to include an additional fifth regime type, the East European, suggested by for example Eikemo et al. in 2008 [22,42,43]. For this, Ferrera’s typology was used and expanded by adding a category composed of the Czech Republic, Hungary, Poland and Slovenia [42]. Estonia and Slovakia are also included in some studies [22,43]. This group of countries has a history with economic instabilities and social reforms during the 1990’s [47] and were argued to form a fifth regime with similar characteristics.

Karim et al.

Karim, Eikemo and Bambra [23] have in 2010 argued that East Asian welfare states also form a cluster of countries and further modify the typology by Eikemo and add a sixth group which includes the East Asian countries (Hong Kong, Japan, Republic of Korea, Singapore and Taiwan). The welfare regimes of the East Asian countries are characterised by low levels of interventions by the government, low investment in social welfare, an underdeveloped provision of public service and a strong reliance on family (e.g. [23]).

Popham

In 2013, Popham [11] used Ferrera’s typology as inspiration and added more countries and extra regimes to the typology. Apart from the typical regime types: Anglo-Saxon; Bismarckian; Nordic; and Southern European, three new regimes are added. These are the Eastern European regime, the Ex-Soviet regime and the Confucian regime.

### Huber and colleagues

The five studies in this group have used different modified versions of the typology by Huber and colleagues and they mainly examine European countries, with analyses based on data from nine countries [24] to 21 countries [48]. Morbidity measures are more common to use. Self-rated health is most common and used in three studies [24,25,48]. Descriptive characteristics of studies in the Huber and colleagues group are shown in Table 4.

**Table 4:** Descriptives of Huber & colleagues group

**Descriptive measure**	**N (author)**
**Data used**	
- OECD, WHO, UN	3 (Navarro*, Chung & Muntaner, Borrell*)
- SHARE	1 (Espelt*)
- ESS	1 (Olsen & Dahl)
- EUROTHINE	1 (Borrell*)
- LIS	1 (Borrell*)
**Health outcome**	
- Self-rated health	3 (Olsen & Dahl, Espelt*, Borrell*)
- Life expectancy	2 (Navarro*, Chung & Muntaner)
- Infant mortality	1 (Navarro*)
- Low birth weight	1 (Chung & Muntaner)
- Longstanding limiting illness	1 (Espelt*)
**Health outcome**	
- Morbidity	6
- Mortality	3
**Measures inequality (total)**	3 (Navarro*, Espelt*, Borrell*)
- SEP (social class)	1 (Espelt*)
- Education	1 (Borrell*)
- Income distribution (Theil index)	1 (Navarro*)

Many articles examined multiple outcomes and hence the number of studies using the different health outcomes (9) is greater than the number of studies (n = 5).

Mortality measures include: life expectancy, infant mortality.

Morbidity measures include: self-rated health, limiting longstanding illness/morbidity/disability, low birth weight.

* = Studies with an inequalities perspective.

Three studies examine population health. Two of these find that health is better in Social democratic countries, one found this for infant mortality and low birth weight [49] and the other for self-rated health [24]. The third found that the Eastern European countries have the lowest levels of self-rated health and no significant differences between the other regimes [48].

Three studies have a health inequality approach. One study finds that inequalities in self-rated health and limiting longstanding illness are found in all three regimes (Social democratic, Christian democratic and Late democratic) but that differences between the social classes are more marked in Late democracies [24]. They also find that education based inequalities in the same health measures are larger in Social democratic countries compared to Christian democratic (for men). Another study also finds that inequalities exist in all regimes (Social democratic, Christian democratic, Liberal and Late democracy) but find gender differences across regimes [25]. For women, inequalities are larger in Social democratic countries compared to Late democracies and for men, inequalities are smaller in Social democratic compared to the three other regimes. Navarro et al. [26] find that Social democratic ideologies tend to implement redistributive policies which reduce social inequalities in health, which perhaps indirectly states that inequalities are smaller in Social democratic regimes. An Additional file shows further information on study characteristics and results [see Additional file 4: Huber and colleagues].

### Theoretical description- Huber and Stephens (and data by Huber, Ragin and Stephens [21])

The typology by Huber and Stephens [20] developed in 2001 is based on political traditions and the allocation of countries is based on the number of years that a country has been governed by a party belonging to a particular political tradition since the 1950’s. The four political traditions are Social democratic (Denmark, Finland, Norway, and Sweden: the most pro-redistributive) Liberal (Canada, Ireland, UK and USA), Christian democratic (Austria, Belgium, France, Germany, Italy, the Netherlands and Switzerland: the least pro-redistributive) and Wage Earners (Australia and New Zealand). Parties in each political tradition display a similar level of commitment to redistributive policies [26].

Different authors have developed this typology further by adding or removing countries or regimes. Navarro, together with different co-authors have removed one regime type, the Wage-Earner, and added another, the ex-Fascist regime or the Late democracies which include Portugal and Spain, and sometimes Greece [24-26]. Other authors have added a fifth regime type consisting of countries in Eastern Europe [48].

### Other typology- Korpi and Palme

Sanders et al. [50] is the only study using this typology [see subsection ‘Theoretical description- Korpi and Palme’]. This study is the only study using oral health as a measure of health. They find that average dental health is better in Finland (representing the Encompassing regime/Social democratic) and worse in Australia (representing the Basic security regime/Liberal). Income-based inequalities were larger in Finland compared to Germany (Corporatist regime). Further information on study characteristics and results can be found in an Additional file [see Additional file 4: Korpi and Palme].

### Theoretical description- Korpi and Palme

In 1998, Korpi and Palme [37] based their typology on the institutional characteristics of welfare states by looking at different countries’ capacity to alleviate income inequality and poverty, specifically examining old age pensions and sickness cash benefits. The classification was based on coverage and generosity and generated five different ideal institutional types characterised as the Basic security, the Corporatist, the Encompassing, the Targeted, and the Voluntary State Subsidised types [38,50].

### Geographical comparisons

The six studies in this group mainly examine differences in health between different European countries or regions and two studies include the United States as a typical Liberal country [51,52]. Countries/regions are not clustered and there is no apparent link to welfare state characteristics. Self-rated health is the most common health measure and is used in four studies [13,52-54] and morbidity measures are generally more common. Descriptive characteristics of studies in the Geographical comparisons group are displayed in Table 5.

**Table 5:** Descriptives of the geographical comparisons group

**Descriptive measure**	**N (author)**
**Data used**	
- SHARE	2 (Ploubidis*, Avendano*)
- OECD, WHO, UN	1 (Granados)
- Register data	1 (Hoffmann*)
- Census	1 (Olafsdottir*)
- Interview survey	1 (Kunst*)
**Health outcome**	
- Self-rated health	4 (Olafsdottir*, Kunst*, Ploubidis*, Avendano*)
- Longstanding limiting illness	1 (Avendano*)
- ≥1 chronic disease.	1 (Avendano*)
- ≥1 activity limitation.	1 (Avendano*)
- Depressive symptoms/depression	1 (Avendano*)
- Infant mortality	1 (Granados)
- Life expectancy	1 (Granados)
- Age specific death rate	1 (Granados)
- Mortality rate	1 (Hoffmann*)
**Health outcome**	
- Morbidity	8
- Mortality	4
**Measures inequality (total)**	5 (Olafsdottir*, Kunst*, Ploubidis*, Avendano*, Hoffmann*)
- SEP (social class)	3 (Olafsdottir*, Kunst*, Hoffmann*)
- Education	1 (Avendano*)
- Gini coefficient	1 (Ploubidis*)

Many articles examined multiple outcomes and hence the number of studies using the different health outcomes (12) is greater than the number of studies (n = 6).

Mortality measures include: life expectancy, infant mortality, mortality rate, age specific death rate.

Morbidity measures include: self-rated health, limiting longstanding illness/morbidity/disability, depression/depressive symptoms, ≥1 chronic disease, ≥1 activity limitation.

* = Studies with an inequalities perspective.

Four studies examine population health and all studies report that the Nordic countries have the best health, but findings differ by various factors. One study finds that young Icelandic people have better self-rated health than American people but that the opposite is found after age 50 [52]. Another finds that although the Nordic countries are still in the lead, the Southern countries are catching up rapidly regarding mortality related measures of health [55]. A third study finds that self-rated health seems to be the best in Sweden, Norway and Denmark but is actually the worst in Finland [53]. Finally, a fourth finds that self-rated health is the best in Social democratic countries compared to other European countries and this effect is largely mediated by more equal income distribution [13].

Five studies have an inequality approach to population health. The results vary in presentation and have different focus points, but four out of five studies point to positive results for Nordic/Social democratic countries. One study finds that the effects of affluence and self-rated health are weaker in Iceland [52]. Another finds that education based inequalities in self-rated health have between the 1980’s and 1990’s remained stable in the Nordic countries but have increased in for example Spain, Italy and the Netherlands [53]. A third uses the Gini coefficient as a measure of inequality and finds that the Nordic countries have the lowest scores which seems to be related to better self-rated health and a higher Gini coefficient score is negatively related to self-rated health [13]. Another study finds that education has more effect on health (morbidity) in Western and Southern Europe and that it is insignificantly related in Northern Europe [54]. Another study finds that inequalities in health are smaller in the United States than in Denmark [51], in contrast to the results of the studies mentioned above which all find positive results for the Nordic countries. An Additional file shows further information on study characteristics and results [see Additional file 4: Geographical comparisons].

### The Regime approach and health

There is great variation in the results presented in the studies when grouped according to what typology they have used, making it problematic to draw generalisable conclusions regarding where population health is better and inequalities in health are the smallest.

The variation in findings across studies applying a regime approach is not possible to understand as a result of the regime typology chosen or the amendments used and we still find a patchy picture with contradictory findings. Nevertheless, since the studies in this category also differ in several other aspects it is still possible that theoretical and empirical differences could account for the diversity in findings.

The studies were initially grouped according to the main outcome; i.e. where health was found to be the best, but no apparent common patterns could be found. Results differed in numerous ways, for example with time, by gender, by measures of population health and health inequalities, making it difficult to draw any conclusions.

The studies were then grouped according to use of health outcome. The studies in this review have used either morbidity- or mortality related measures and although these are both valid measures of health, they might give different results. The two big groups that are classified as mortality related measures are life expectancy and infant mortality. The studies that look at life expectancy find that East Asian countries have higher life expectancy than other regimes. The studies that examine infant mortality find that the Nordic countries have the lowest rates of infant mortality. Few studies examine inequalities and do not give any clear results.

Studies that look at morbidity measures such as self-rated health find mixed results. Some find that the Nordic countries have better self-rated health while others find that other regimes have better health. No consensus regarding which regime has the best health can be found; some find that Liberal countries have better health than Conservative, and others find the opposite. No clear pattern is seen for inequalities in self-rated health; there is no consensus of which regime has the smallest.

The studies were then listed according to the number of countries of which the studies are based but no apparent pattern could be found.

Finally, the studies were grouped according to the type of data used. All studies using ESS data, except for one, which does not find any significant differences between the typologies, find that other countries, and not the Nordic countries, have the best health. Most of these studies use self-rated health as health outcome. It seems as though other countries have smaller inequalities compared to the Nordic countries. Most of these studies are from one main group of authors, namely Bambra and Eikemo, and most look at Scandinavian/Social democratic, Conservative, Liberal, Southern and Eastern European countries. Most studies using OECD data conclude that Nordic countries have better health. Most of these use infant mortality as a measure of health. Regarding inequalities in health, it is difficult to draw any conclusions.

### Institutional approach

The Institutional approach is the second largest of the three groups with 14 studies fitting the criteria. Five studies use a health inequality perspective, examining inequalities by socioeconomic position/status, type of mother (lone vs. coupled), education and income. The studies have been classified according to main type of policy area: family [15,16,56-59], pensions [17,59-61], economic assistance and unemployment benefits [58,62,63] and access to health care [64-66]. Two articles cover several policy areas [58,59]. The selected studies together use 12 different health measures (13 if including ‘immunisation’), some use several and some only one. It is more common to use mortality measures (used 15 times) as health indicator than it is morbidity measures (used 10 times). Descriptive characteristics of studies with an Institutional approach can be found in Table 6.

**Table 6:** Descriptive characteristics of the studies with an institutional approach (n = 14)

**Descriptive measure**	**n (%)**	Author (health inequality perspective = *)
**Year of publication**		
2005	1 (7%)	Tanaka*
2006	2 (14%)	Borrell*, Nordenmark
2007	3 (21%)	Fritzell*, James*, Korda*
2008	1 (7%)	Lundberg et al.
2009	---	---
2010	6 (43%)	Burstrom et al.*, Esser & Palme, Ferrarini & Norström, Ferrarini & Sjoberg, Kangas, Norstrom & Palme
2011	1 (7%)	Engster & Olofsdotter Stensota
2012	---	---
2013	---	---
**Articles using health inequality approach**	6 (43%)	
**Health inequality measure**		
Type of mother (lone vs. coupled)	2 (33%)	Fritzell et al.*, Burstrom et al.*
Socioeconomic position/status	1 (17%)	Korda et al.*
Income	1 (17%)	James et al.*
Education	1 (17%)	Borrell et al.*
**Type of benefit/policy**		
Family benefits	6 (43%)	Burstrom et al.*, Engster & Olofsdotter Stensota, Ferrarini & Norstrom, Ferrarini & Sjoberg, Lundberg et al., Tanaka
Pension benefits	4 (29%)	Esser & Palme, Kangas, Lundberg et al., Norstrom & Palme
Economic assistance and unemployment benefits	3 (21%)	Ferrarini & Sjoberg, Fritzell et al.*, Nordenmark et al.
Access to health care	3 (21%)	Borrell et al.*, James et al.*, Korda et al.*
**Health outcomes**		
Infant mortality	4 (29%)	Ferrarini & Norstrom, Ferrarini & Sjoberg, Lundberg et al., Tanaka
Self-rated health	4 (29%)	Burstrom et al.*, Esser & Palme, Ferrarini & Sjoberg, Fritzell et al.*
Mortality rate	3 (21%)	Fritzell et al.*, James et al.*, Korda et al.*
Limiting longstanding illness/morbidity/disability	2 (14%)	Burstrom et al.*, Fritzell et al.*
Old-age excess mortality	2 (14%)	Lundberg et al., Norstrom & Palme
Child mortality	2 (14%)	Engster & Olofsdotter Stensota, Tanaka
Life expectancy at birth	1 (7%)	Kangas
Change in life expectancy at birth	1 (7%)	Kangas
AIDS mortality	1 (7%)	Borrell et al.*
Hospitalisation	1 (7%)	Fritzell et al.*
Psychological distress in GHQ (General Health Questionnaire)	1 (7%)	Nordenmark et al.
Low birth weight	1 (7%)	Tanaka
(Immunisation)	1 (7%)	Tanaka
**Health outcome**		
Mortality measure		15
Morbidity measure		10

Many articles examined multiple outcomes and hence the number of studies using the different health outcomes (25) is greater than the number of studies (n = 14).

Mortality measures include: infant mortality, life expectancy at birth, mortality rate, old-age excess mortality, child mortality, AIDS mortality, and change in life expectancy at birth.

Morbidity measures include: self-rated health, limiting longstanding illness/morbidity/disability, hospitalisation, psychological distress in GHQ, low birth weight.

* = Studies with an inequalities perspective.

### Family benefits

There is general consensus that generous family benefits and the dual-earner family policy model are beneficial for health, both for adults’ self-rated health and also child mortality. Universal family policies seem to be beneficial for all, not only those who use it. One study [15] looks at inequalities in health and uses ‘type of mother’ as measure of stratification. They find that generous family policies provide protection from poor health, poverty and unemployment to mothers in general and particularly to lone mothers. An Additional file shows further information on study characteristics and results [see Additional file 5: Family benefits].

### Pension benefits

There seems to be general agreement of generous pensions being related to better health and higher life expectancy. Most studies suggest that basic security pensions are associated with lower old age excess mortality [59,61] and a higher life expectancy [17]. There is less evidence supporting income security pensions’ effect on health. The different pension benefits perhaps work differently for men and women: income security pensions seem more important for men’s health and basic security pensions seem more important for women’s [60]. For further information on study characteristics and results see an Additional file [see Additional file 5: Pension benefits].

### Economic assistance and unemployment benefits

Universal systems of economic assistance [62] and unemployment benefits [58,63] seem to be associated with a healthier population. This seems to apply to the whole population, not only to the health of the unemployed [58]. An Additional file shows further information on study characteristics and results [see Additional file 5: Economic assistance and unemployment benefits].

### Access to health care

Absolute inequalities in mortality by socioeconomic status (income and education) seem to decrease with universal health care. However, the relative gap seems to increase, i.e. advantaged people obtain disproportionate benefits of health care, and access, or perhaps adherence to health care seems lower for people in lower socioeconomic groups [64,66]. For further information on study characteristics and results [see Additional file 5: Access to health care].

### The institutional approach and health

Most studies in this approach seem to agree that generous policies and benefits are associated with health in a positive way for all people in a population, not only those who are directly affected or targeted and receive the actual benefit.

### Expenditure approach

The Expenditure approach is the smallest of the three approaches, only eight studies fit the criteria. There is perhaps an increased tendency of using this approach for cross-country comparisons of population health (see Additional file 2 for detailed timelines of publication year). Two studies use a health inequality perspective and both examine inequalities by education and were published in 2012. One study covers both social and health spending, three studies cover social spending only, and four cover health spending only. The selected studies use various health measures, nine different in total. Some studies analyse several health measures and others look at only one. By categorising the health outcomes into either an outcome related to mortality or morbidity, one finds that different mortality outcomes are the most common (11 compared to 4). Descriptive characteristics of studies with an Expenditure approach are shown in Table 7.

**Table 7:** Descriptive characteristics of the studies with an expenditure approach (n = 8)

**Descriptive measure**	**n (%)**	Author (health inequality perspective = *)
**Year of publication**		
2005	---	---
2006	---	---
2007	2 (25%)	Olsen & Dahl, Wu & Chiang
2008	---	---
2009	---	---
2010	2 (25%)	Kangas, Stuckler et al.
2011	1 (13%)	Bradley
2012	3 (38%)	Dahl & van der Wel*, Gesthuizen et al.* Vavken et al.
2013	---	---
**Articles using health inequality approach**	2 (25%)	
**Health inequality measure**	2 (100%)	Dahl & van der Wel, Gesthuizen et al.
Education		
**Type of expenditure**		
Social spending	4 (50%)	Bradley et al., Dahl & van der Wel*, Kangas, Stuckler et al.
Health spending	5 (63%)	Bradley et al., Gesthuizen et al.*, Olsen & Dahl, Vavken et al., Wu & Chiang
**Health outcomes**		
Self-rated health	3 (38%)	Dahl & van der Wel*, Gesthuizen et al.*, Olsen & Dahl
Infant mortality	2 (25%)	Bradley et al., Wu & Chiang
Life expectancy at birth	2 (25%)	Bradley et al., Kangas
Mortality rate	2 (25%)	Stuckler et al., Vavken et al.
Potential years of life lost	2 (25%)	Bradley et al., Vavken et al.
Child mortality (under 5 mortality rate)	1 (25%)	Wu &Chiang
Low birth weight	1 (13%)	Bradley et al.
Maternal mortality	1 (13%)	Bradley et al.
Change in life expectancy at birth	1 (13%)	Kangas
**Health outcome**		
Mortality measure		11
Morbidity measure		4

Many articles examined multiple outcomes and hence the number of studies using the different health outcomes (15) is greater than the number of studies.

Mortality measures include: infant mortality, life expectancy at birth, mortality rate, potential years of life lost, child mortality, maternal mortality, change in life expectancy at birth.

Morbidity measures include: self-rated health, low birth weight.

* = Studies with an inequalities perspective.

### Health spending

Some studies find that health spending is associated with life expectancy and maternal mortality [67], general mortality and a reduction of life years lost [68], and lower infant mortality rates [69]. One study finds that social spending on health is negatively correlated with health for women and unrelated for men [48]. The authors suggest that a reason for this might be that additional spending on health might have little effect on OECD countries since expenditure levels are already high in many of these countries. One study [70] looks at inequalities and finds that in countries where the government spends a lot of money on healthcare (and has a highly modernised labour market) the relative risk of lower educated people being in poor health is smaller. An Additional file shows further information on study characteristics and results [see Additional file 6: Health spending].

### Social spending

Two studies find that social spending is associated with life expectancy, infant mortality, potential years of life lost [67], and mortality [71]. One study [17] finds conflicting results; the relationship between social spending and life expectancy vary from cross-section to cross-section. The study finds that initial investment in social policy leads to increases in life expectancy but after a certain level of spending, the extra spending does not contribute that much. The study looking at health inequalities [5] finds that social spending seems to be associated with lower education based inequalities in health among women and, to a lesser degree, among men. Additionally, those with primary education benefit more from high social transfers than those with tertiary education. For further information on study characteristics and results see an Additional file [see Additional file 6: Social spending].

### The expenditure approach and health

Most studies in the Expenditure approach agree that social and health spending is associated with increased levels of health in one way or the other. The studies that do not find these positive associations do not see consistent findings over time. These studies also show evidence that after a certain level of spending additional spending does not contribute that much, showing a curvilinear association. Both studies with the inequality perspective find that spending is beneficial for those with lower educational status. Both studies examine self-rated health and therefore no conclusions can be drawn regarding mortality (which is the most common use of measure in this approach).

## Discussion

The starting point for this review has been the mixed and contradictory findings arising from research on welfare state characteristics and health and health inequalities. These contradictions either suggest that 1) policies directed at the causes of the causes are much less important for health and health inequalities than we have been assuming, or 2) there are fundamental theoretical and/or empirical shortcomings in many studies in this field. This area evidently needs to be further explored in order to fully understand the inconsistent results and is of importance not only to welfare research but also to epidemiology. The results in this review add an important piece to the puzzle by clarifying and describing why previous studies have not been able to come to unequivocal conclusions.

Our analytical approach has been to sort the relevant studies found according to their approach to measure welfare state characteristics, something that to our knowledge, has not been done before. Of the three main types (regime, institutional and expenditure), the Regime approach is by far the most common. However, while the fundamental approach is the same for these studies we find large variations in the theoretical basis as well as the countries and regime types included. In Esping-Andersen’s original work [19] several clusters of countries are actually being suggested based on de-commodification, social stratification, and the private-public mix of social provision, respectively. Most followers are using de-commodification as their starting point. In addition, many studies in this public health field that employ Esping-Andersen’s work make amendments of clusters and countries and the theoretical underpinning is therefore not as strong as often assumed.

However, even when we sort studies according to the regime theory employed and the amendments made to these, results are diverse and contradictory. Hence, it is not inconsistencies between different theories or different empirical applications of these that is the only or main problem, but a more general problem with welfare state regimes when applied to outcomes such as health and health inequalities.

A further problem is that different health measures are used, which adds to the complexity of drawing conclusions about where health is the best and health inequalities the smallest since choice of measure will highly affect the outcome and the conclusions drawn. When stratifying our material according to type of health measure used some consistent results can be found regarding levels of mortality. Morbidity related measures show mixed results and may reflect data and reporting problems. However, in search for consistencies regarding health inequalities, not much added clarity is achieved.

Many researchers in comparative welfare regime and health research agree that welfare states cluster together into certain regimes. However, there is less agreement about which typology to apply and when, and this therefore remains an open issue. Since there is no total agreement about which typology to use, several classifications have emerged, many of which are rather similar and overlap each other, all intending to capture the essence of a welfare state. These typologies have sometimes emerged on unclear grounds, for example, it seems as though some have emerged based on the country data available to each author and not on strong theoretical grounds. Interpretations and comparisons of findings from these studies will be complicated by typologies that have been constructed differently and further used as an independent variable aiming to ‘explain’ variations in health and health inequalities across countries. By adding a regime such as the Eastern European regime, the picture becomes more complete, but it also becomes more complex and this tends to change the whole focus of the study. These studies tend to find that Central and Eastern European countries fare the worst. The health situation in former communist countries is an important and complicated issue in its own right. However, while this is likely to be linked to social and policy factors it is questionable if the addition of these countries to the existing and already conflicting research is especially helpful.

This field of research has a long history of debate. Many critics have pointed out that there are problems with typologising. One of the most outspoken is Baldwin [72], who in 1996 critically wrote that two countries in a regime cluster can be inconsistent among policy areas, and that welfare state studies have “exhausted its explanatory power and is no longer bearing fruit”. Kasza [73], is another who in 2003 concluded that “few national welfare systems are likely to exhibit the internal consistency necessary to validate the regime concept, and that policy-specific comparisons may be a more promising avenue for comparative research”. Mackenbach [74] is more recent and writes in 2012 that Esping-Andersen’s typology is not “suitable for distinguishing countries with different types of health care provision. Generousness or universalism in other parts of the welfare state, e.g. for income support, does not appear to predict generousness or universalism of health care provision”. The UK for example, is usually placed in the Liberal regime group, but at the same time, it has a universal health system free of charge. Mackenbach [74] suggests “that if we want to study the health impacts of welfare arrangements we might better not take Esping-Andersen’s classification as starting point”. On the other hand, many researchers refer to Southern, Northern and other groups of countries in a way that suggest an underlying idea about fundamental commonalities in those groups (including Mackenbach).

With time comes change, and all is well if all countries in each welfare state cluster move forward together, but this is seldom the case. Countries will have different experiences and might well move in different directions at different paces. A country can with time go through policy changes in eligibility, structures or financing that could technically and potentially reposition it from one regime cluster to another. Although these transformations might be “work in progress”, quite a few researchers agree that countries and their politics change with time. For example, Kvist et al. [75] concluded that the small changes found across policies for families with children, for the unemployed, for the ill, and for the older in the 2000s, when added together, challenge the concept of the Nordic welfare policies. Kuivalainen and Nelson [76] find that the social assistance in the Nordic countries is moving closer to some of the features and outcomes of other regimes in terms of benefit generosity and poverty outcomes. They conclude that the Nordic social assistance classification into a separate model of social welfare is not as distinct as it was 20 years ago. A recent OECD report about income inequalities finds that in Sweden, many times seen as the archetypical Nordic country, the relative income poverty rate has increased the most during the last 20 years and particularly among children and youth. In other words, Sweden’s capacity to protect the vulnerable groups against poverty has been weakened due to an inability to keep up with the increases in general income [77]. If this continues the Nordic countries might experience poverty rates similar to those in several Liberal and Conservative welfare states meaning that one of the most significant features of the Nordic welfare states will disappear [76].

Hence, our first conclusion based on earlier reviews and our own attempts to analyse the reasons for diverging and conflicting findings from the growing body of research on welfare state characteristics and health/health inequalities is that the welfare state Regime approach is not a fruitful way forward. We do not assert that this can be extended as a general conclusion. Rather, the Regime approach has been important for welfare state research, and especially so perhaps for analyses of the welfare state as a dependent variable. It can also be highly informative for descriptive purposes. But as a tool for analyses of how policies and institutions that impact on the wider social determinants of health actually affect health inequalities, it is simply too crude and imprecise. When adding the fact that few studies in practice adopt the same Regime approach (although many use the same labels), there is no wonder that the results produced are diverging and even conflicting.

In recent years there has been a theoretical and methodological development of welfare state models and regimes, where both the ‘productive’ and ‘protective’ dimensions of welfare state activities are included [78,79]. This allows for a more complex categorisation of strengths and weaknesses in different countries where both education and active labour market programmes (‘productive’), and employment and income protection (‘protective’) policies are considered. To our knowledge this has not yet been applied to health and health inequalities outcomes, but given that this approach produces a more nuanced picture where also countries outside the traditional OECD countries can be included [80], it seems more promising than more traditional ways of clustering countries. Another development in comparative welfare research is the “Varieties of Capitalism” approach [81] which compares countries based on type of capitalism. Both of these do, however, still represent a clustering of countries, and although they are likely to be more promising than the different ideal types typically employed in the studies included in our review, several of the caveats are likely to apply.

In contrast to regime types, the Institutional and Expenditure approach focus on the more specific “welfare outputs” delivered by the welfare state, either captured as the formal legislated rights that people have or the money spent on the programme. Hence, the approaches are much more able to study specific policies but can easily also study the total effort by combining different rights/spendings. Another major difference between these two approaches and the Regime approach is that where the Regime approach has to rely on country cluster average differences, the Institutional and Expenditure approaches give us a variable approach. This means that we can allow for countries to differ in their policies in different areas of interest (social protection, family policies, health care policies, labour market policies etc.). This, in turn, is likely to increase the policy relevance of studies as well as our understanding of the processes involved when health inequalities are generated.

While the Institutional and Expenditure approaches are more promising in principle for health inequality research, there are to date a limited number of studies of this kind. Yet, the clear impression from taking these studies combined is still that more social spending and more generous social rights lines up with lower mortality, better health and, probably, smaller health inequalities. We would like to see more studies using these approaches, but on basis of the ones that exist it is tempting to suggest a solution to the ‘Nordic paradox’; while welfare policies of a ‘Nordic’ kind is indeed promoting better health and smaller health inequalities, it not the case that such policies are mainly found in the Nordic countries. When looking at country clusters, ‘better’ and ‘worse’ policies for public health and health inequalities are found in many clusters, and the results becomes highly dependent on the countries actually included. When looking directly at institutional social rights or social spending the relations between policies and health outcomes becomes uncovered in a much clearer way.

It is important to stress that the Institutional approach to a large extent was formulated as a critique of the Expenditure approach. The latter has been accused of being faulty since it does not address two of the main features of a welfare state; social citizenship and social rights. There have also been doubts regarding that high spending means nothing more than extensive social problems. The level of spending might therefore not tell us much about the characteristics of a welfare state’s social or health programmes. Kangas and Palme [8] find that the advanced rich countries seem to use roughly the same amount of their GDP on welfare. Even though spending levels are similar, the distributional consequences can be greatly divergent. However, this critique has also led to adaptations and procedures to take differences in need into account, thereby closing the gap between the two approaches in empirical terms at least [5,82].

But, the Institutional approach focusing on legislated social rights has shortcomings too. It tends to capture the principles for certain type cases, while the lived experience of people in need of social protection can be something else. It might also be important to include several dimensions of social rights, like coverage and replacement rates, to get a balanced picture. In contrast to spending data, such data on the legislated rights are not produced routinely but requires large efforts to collect. Hence, there are weaknesses and strengths to both approaches, and in relation to outcomes like health inequalities, they are likely to be complementary rather than mutually exclusive.

### Limitations

This review was based on empirical studies published in peer-reviewed journals. There is a small risk that other studies of welfare states and health inequalities are to be found in e.g. the grey literature, but if so these have been overlooked.

We have set the starting point for the literature search to 2005 and relevant studies prior to this year have been missed in our search. However, we started off by revising the three large reviews [3,4,9], which are based partly on studies prior to 2005, and their results are also somewhat inconsistent regarding welfare research and health inequalities. This indicates that studies prior to 2005 would not contribute much to the overall picture. In addition, the Publication timelines [see Additional file 2] indicate that the number of studies increases over time, which means that the risk that we have missed important studies prior to 2005 is small.

Most importantly, however, it is necessary to notice that the three approaches identified are unbalanced; the Regime approach is by far the largest. While this means that our conclusions regarding the Regime approach are fairly well underpinned, conclusions regarding the merits of the Institutional and Expenditure approaches are based on a small number of studies. While this reflects the reality, it is important to keep in mind when evaluating our conclusions. For example, if more studies are produced using these two approaches it may well be that less consistent results emerge also for them.

## Conclusions

The wider social determinants of health, the causes of the causes, are of great importance for health and well-being, and the collective resources in terms of social protections and services provided by the welfare state are likely to be more important for those that have fewer resources in their own control. From this follows that a range of welfare state policies are important for health and health inequalities, but the question is how we best can study this in more detail. Most likely, there is not one answer to that question. However, earlier reviews and our own attempts to find some consistency strongly suggest that further studies of the Welfare Regime approach and health inequalities do not seem to lead us much further.

Instead, we will need a multitude of different types of studies, including larger analyses of social spending and social rights in various policy areas and how these are linked to health in different social strata. But, we also need more detailed evaluation of specific programmes or interventions, as well as more qualitative analyses of the experiences of different types of policies among the people and families that need to draw on the collective resources. There are many roads that will take us forward towards a better understanding of how health inequalities are generated and how policies directed to the social determinants of health can prevent or amplify these processes.

## Competing interests

The authors declare that they have no competing interests.

## Authors’ contributions

KB, OL and MÅY all made important contributions to the manuscript. KB had the main responsibility for drafting the manuscript. OL and MÅY contributed substantially to the design of the study. KB conducted the literature searches, screened all potentially relevant studies as part of the preliminary inclusion/exclusion and reviewed papers obtained for inclusion, wrote the methodology and results section, and part of the background and discussion. OL wrote part of the introduction and discussion. OL and MÅY reviewed papers for which there was disagreement as to whether they should be included. All authors participated in discussions regarding methodology and findings, and reviewed drafts of the manuscript and approved the final version.

## Pre-publication history

The pre-publication history for this paper can be accessed here:

http://www.biomedcentral.com/1471-2458/13/1234/prepub

## Supplementary Material

Additional file 1 **Descriptive characteristics of the studies with a regime approach (n = 34).** This file contains a table with descriptive characteristics of the articles included in the Regime approach. It includes details of publication year, countries under study, health inequality measures, health outcome variables, and number of times each typology group has been used. Click here for file

Additional file 2 **Timelines of publication year.** This file contains four timeline figures of publication year of the selected studies. The first shows all studies included in the review, the remaining timelines display the publication dates of the studies selected for the three main approaches to comparative welfare research; the Regime approach, the Institutional approach, and the Expenditure approach. Click here for file

Additional file 3 **Table of the different typologies used in the studies included in this review.** This file contains a table illustrating the different welfare regime typologies used in the studies. It shows the different clusters, which countries are included in each, and the number of studies that have adopted each. Click here for file

Additional file 4 **Tables of studies used in the review sorted by welfare regime typology.** The tables in this file illustrate the data used, the number of countries looked at, health outcome/s, measure of health inequality, typology and main results for each of the main groups; Esping-Andersen, Ferrera, Huber and colleagues, Korpi and Palme and Regional comparisons. Click here for file

Additional file 5 **Tables of studies used in the review sorted by Institutional approach.** The tables in this file illustrate the different institutional approaches used in the studies. They are sorted by type of policy (pension benefits, economic assistance and unemployment benefits, family benefits, and access to health care), the data used, health outcome/s, measure of health inequality, and main results for each of the main groups. Click here for file

Additional file 6 **Tables of studies used in the review sorted by Expenditure approach.** The tables in this file illustrate the two Expenditure approaches used in the studies. They are sorted by type of spending (health spending or social spending), the data used, health outcome/s, measure of health inequality, and main results for each of the main groups. Click here for file
