# Supplementary material for: Understanding the role of welfare state characteristics for health and inequalities – an analytical review
Source: BMC Public Health. 2013 Dec 27;13:1234. doi: 10.1186/1471-2458-13-1234 (PMC3909317; doi:10.1186/1471-2458-13-1234)
Supplement: Additional file 1 — Descriptive characteristics of the studies with a regime approach (n = 34). This file contains a table with descriptive characteristics of the articles included in the Regime approach. It includes details of publication year, countries under study, health inequality measures, health outcome variables, and number of times each typology group has been used. [file 1471-2458-13-1234-S1.docx]

**Additional file 1. Descriptive characteristics of the studies with a regime approach, n=34.**

| **Descriptive measure** | **n (%)** | **Author (health inequality perspective = *)** |
| --- | --- | --- |
| **Year of publication**  2005  2006  2007  2008  2009  2010  2011  2012  2013 | 1  3  4  4  6  6  2  7  1 | Kunst et al*  Bambra, Navarro et al*, Zamvbon et al*  Chung & Muntaner, Olafsdottir*, Olsen & Dahl, Rostila  Eikemo et al*x2, Eikemo et al, Espelt et al*  Avendano et al*, Bambra & Eikemo*, Bambra et al*, Borrell et al*, Sanders et al*, Sekine et al*  Bambra et al*, Dragano et al, Esser & Palme, Granados, Huijts et al, Kangas, Karim et al  Hoffmann*, Sacker et al*  Chuang et al, Deeming & Hayes, Harding et al*, Ploubidis et al*, Raphael, Richter et al*, van der Wel et al*  Popham et al* |
| **Countries under study**  1-10  11-20  21-30  31+ | 10 (9)  11(5)  9 (4)  4 (3) |  |
| **Articles using health inequality approach**  **Health inequality measure**  Socioeconomic position/status/  social class  Education  Income  Employment status  Popham measure  Gini coefficient  Income distribution (Theil index) | 21 (62%)  8 (38%)  6 (29% )  2 (10%)  2 (10%)  1 (5%)  1 (5%)  1 (5%) | Espelt et al, Hoffmann, Kunst et al, Olafsdottir, Richter et al, Sacker et al, Sekine et al, Zambon et al  Avendano et al, Bambra et alx2, Borrell et al, Eikemo et al, van der Wel  Eikemo et al, Sanders et al  Bambra & Eikemo, Harding et al  Popham et al  Ploubidis et al  Navarro et al |
| **Health outcomes**  Self-rated health  Life expectancy  Limiting longstanding illness/morbidity/  disability  Infant mortality  Mortality rate  Depression/depressive symptoms  Low birth weight  Change in life expectancy at birth  ≥1 chronic disease  ≥1 activity limitation  Self-reported happiness  Psychosocial quality of work  Health complaints  Oral health  Physical and mental health functioning  General wellbeing  Health behaviours  Health symptom load  **Health outcome**  Morbidity measure  Mortality measure | 18 (53%)  8 (24%)  7 (21%)  7 (21%)  5 (15% )  1 (3%)  1 (3%)  1 (3%)  1 (3%)  1 (3%)  1 (3%)  1 (3%)  1 (3%)  1 (3%)  1 (3%)  1 (3%)  1 (3%)  1 (3%) | Avendano*, Bambra et al*, Bambra et al*, Bambra & Eikemo*, Borrell et al*, Eikemo et al*x2, Eikemo et al, Espelt et al*, Huijts et al, Kunst et al*, Olsen & Dahl, Olafsdottir *, Ploubidis et al*, Richter et al*, Rostila, Sacker et al*, Zambon*  Chuang et al, Granados, Kangas*, Karim et al, Navarro et al*, Popham et al*, Raphael, Rostila  Avendano*, Bambra et al*, Bambra & Eikemo*, Eikemo et al*x2, Espelt et al*, Van der Wel et al*  Bambra, Chuang et al, Chung & Muntaner, Granados, Karim et al, Navarro et al*, Raphael  Dragano et al, Granados, Harding et al*, Hoffmann*, Popham et al*  Avendano*  Chung & Muntaner  Kangas*  Avendano*  Avendano*  Deeming & Hayes  Dragano et al  Richter et al*  Sanders et al*  Sekine et al*  Zambon*  Zambon*  Zambon*  32  18 |
| **Typologies based on**  Ferrera  Esping-Andersen  Huber and colleagues  Other- Korpi and Palme  **Geographical comparisons** | 13 (38%)  9 (26%)  5 (15%)  1 (3%)  6 (18%) | Bambra et al*, Bambra et al*, Bambra & Eikemo*, Chuang et al, Dragano et al, Eikemo et al*x2, Eikemo et al, Huijts et al, Karim et al, Popham et al*, Richter et al*, Van der Wel et al*  Bambra, Deeming & Hayes, Harding et al*, Kangas, Raphael, Rostila, Sekine et al*. Sacker et al*, Zambon et al*  Borrell et al*, Chung & Muntaner, Espelt et al*, Navarro et al*, Olsen & Dahl  Sanders et al*  Avendano et al*, Granados, Hoffmann*, Kunst et al*, Olafsdottir*, Ploubidis et al* |

Many articles examined multiple outcomes and hence the number of studies using the different health outcomes (50) is greater than the number of studies (n=34).

Mortality measures include: life expectancy, infant mortality, mortality rate, change in life expectancy at birth.

Morbidity measures include: self-rated health, limiting longstanding illness/morbidity/disability, depression/depressive symptoms, low birth weight, ≥1 chronic disease, ≥1 activity limitation, self-reported happiness, psychosocial quality of work, health complaints, oral health, physical and mental health functioning, general wellbeing, health behaviours, health symptom load.
