# Supplementary material for: Understanding the role of welfare state characteristics for health and inequalities – an analytical review
Source: BMC Public Health. 2013 Dec 27;13:1234. doi: 10.1186/1471-2458-13-1234 (PMC3909317; doi:10.1186/1471-2458-13-1234)
Supplement: Additional file 2 — Timelines of publication year. This file contains four timeline figures of publication year of the selected studies. The first shows all studies included in the review, the remaining timelines display the publication dates of the studies selected for the three main approaches to comparative welfare research; the Regime approach, the Institutional approach, and the Expenditure approach. [file 1471-2458-13-1234-S2.docx]

# Additional file 2. Timelines of publication year.

**All studies in the review (inequalities’ studies are marked with an asterisk*).**

| 2005 | 2006 | 2007 | 2008 | 2009 | 2010 | 2011 | 2012 | 2013 |
| --- | --- | --- | --- | --- | --- | --- | --- | --- |

Kunst et al.*

Tanaka

Bambra

Borell et al.*

Navarro et al.*

Nordenmark et al.

Zambon et al*.

Chung & Muntaner

Fritzell et al.*

James et al.*

Korda et al.*

Olafsdottir*

Olsen & Dahl

Rostila

Wu & Chiang

Eikemo et al.*

Eikemo et al.*

Eikemo et al.

Espelt et al.*

Lundberg et al.

Avendano et al.*

Bambra & Eikemo*

Bambra et al.*

Borell et al.*

Sanders et al.*

Sekine et al.*

Bambra et al.*

Burström et al.*

Dragano et al.

Esser & Palme

Ferrarini and Norstom

Ferrrarini & Sjoberg

Granados

Huijts et al.

Kangas

Karim et al.

Norstrom & Palme

Stuckler et al.

Bradley et al.

Engster & Olofsdotter Stensota

Hoffmann*

Sacker et al.*

Chuang et al.

Dahl & van der Wel*

Deeming & Hayes

Gesthuizen et al.*

Harding et al.*

Ploubidis et al.*

Raphael

Richter et al.*

Vavken et al

van der Wel et al*.

 Popham et al.*

Popham et al.*

**All studies in the ‘Regime approach’ (inequalities’ studies are marked with an asterisk*).**

| 2005 | 2006 | 2007 | 2008 | 2009 | 2010 | 2011 | 2012 | 2013 |
| --- | --- | --- | --- | --- | --- | --- | --- | --- |

Kunst et al*

Bambra

Navarro et al.*

Zambon et al*.

Chung & Muntaner

Olafsdottir*

Olsen & Dahl

Rostila

Eikemo et al.*

Eikemo et al.*

Eikemo et al.

Espelt et al.*

Avendano et al.*

Bambra & Eikemo*

Bambra et al.*

Borrell et al.*

Sanders et al.*

Sekine et al.*

Bambra et al.*

Dragano et al.

Esser & Palme

Granados

Huijts et al.

Kangas

Karim et al.

Hoffmann*

Sacker et al.*

Chuang et al.

Deeming & Hayes

Harding et al.*

Ploubidis et al.*

Raphael

Richter et al.*

van der Wel et al*.

 Popham et al.*

Popham et al.*

**All studies in the ‘Institutional approach’ (inequalities’ studies are marked with an asterisk*).**

| 2005 | 2006 | 2007 | 2008 | 2009 | 2010 | 2011 | 2012 | 2013 |
| --- | --- | --- | --- | --- | --- | --- | --- | --- |

Tanaka

Fritzell et al.*

James et al.*

Korda et al.*

Borell et al.*

Nordenmark et al.

Lundberg et al.

Burström et al.*

Esser & Palme

Ferrarini & Norstom

Ferrrarini & Sjoberg

Kangas

Norstrom & Palme

Engster & Olofsdotter Stensota

Popham et al.*

**All studies in the ‘Expenditure approach’ (inequalities’ studies are marked with an asterisk*)**

2005 2006 2007 2008 2009 2010 2011 2012 2013

Olsen & Dahl

Wu & Chiang

Kangas

Stuckler et al.

Bradley et al.

Dahl & van der Wel*

Gesthuizen et al.*

Vavken et al

Popham et al.*
