# Supplementary material for: Understanding the role of welfare state characteristics for health and inequalities – an analytical review
Source: BMC Public Health. 2013 Dec 27;13:1234. doi: 10.1186/1471-2458-13-1234 (PMC3909317; doi:10.1186/1471-2458-13-1234)
Supplement: Additional file 3 — Table of the different typologies used in the studies included in this review. This file contains a table illustrating the different welfare regime typologies used in the studies. It shows the different clusters, which countries are included in each, and the number of studies that have adopted each. [file 1471-2458-13-1234-S3.docx]

**Additional file 3. Table of the different typologies used in the studies included in this review.**

| **Author** | **Measures** | **Welfare state regimes** | | | | | | | **# used** |
| --- | --- | --- | --- | --- | --- | --- | --- | --- | --- |
| Esping-Andersen (1990) | 18 countries   - Decommodification - Social stratification - Private-public mix | **Liberal**  Australia  Canada  Ireland  New Zealand  UK  USA | **Conservative**  Finland  France  Germany  Italy  Japan  Switzerland | **Social democratic** Austria  Belgium  Denmark  The Netherlands  Norway  Sweden |  |  | | | 4  Harding et al, Sekine et al, Kangas, Bambra, |
| Castles and Mitchell (1993) | 14 countries   - Aggregate welfare expenditure - Benefit equality | **Liberal**  Ireland  Japan  Switzerland  USA | **Conservative**  Germany  Italy  Netherlands | **Non-right hegemony**  Belgium  Denmark  Norway  Sweden | **Radical**  Australia  New Zealand  UK |  | | | 2  Sacker et al, Deeming and Hayes |
| Ferrera (1996) | 15 countries   - Coverage - Replacement rates - Poverty rates | **Anglo-Saxon**  Ireland  UK | **Bismarckian**  Austria  Belgium  France  Germany  Luxembourg  The Netherlands  Switzerland | **Scandinavian**  Denmark  Finland  Norway  Sweden |  | **Southern**  Italy  Greece  Portugal  Spain | | | 3  Dragano et al, Bambra et al, Bambra et al |
| Korpi and Palme (1998) | 18 countries   - Social expenditure as % GDP - Luxembourg income study - Institutional characteristics | **Basic security**  Canada  Denmark  Ireland  The Netherlands  New Zealand Switzerland  UK  USA | **Corporatist**  Austria  Belgium  France  Germany  Italy  Japan | **Encompassing**  Finland  Norway  Sweden | **Targeted**  Australia | **Voluntary state**  **subsidised**  No countries  were fitted | | | 1  (Sanders et al) |
| Navarro, Schmitt, and Astudillo ([2004](#_ENREF_52)) | 13 countries  Based on Huber and Stephens (2001)   - Based on political tradition | **Liberal**  England  Ireland | **Christian democratic**  Belgium  France  Germany  Italy  The Netherlands | **Social democratic**  Denmark  Finland  Norway  Sweden |  | **Late**  **democracies**  Portugal  Spain | | | 1  (Borell) |
| Navarro (2006) | 17 countries  Based on Huber and Stephens (2001)   - Based on political tradition | **Liberal**  Canada  Ireland  UK  USA | **Christian democratic**  Belgium  France  Germany  Italy  The Netherlands | **Social democratic**  Austria  Denmark  Finland  Norway  Sweden |  | **Former**  **dictatorships**  Greece  Portugal  Spain | | | 1  (Navarro) |
| Zambon et al (2006) | 31 countries  Based on Esping-Andersen (1990) | **Liberal**  Australia  Canada  Ireland  Israel  New Zealand  UK  USA | **Conservative**  Belgium  France  Germany  Japan  the Netherlands | **Social Democratic**  Austria  Denmark  Finland  Norway  Sweden |  | **Mediterranean/Southern**  Greece  Italy  Portugal  Spain | **Eastern/post-Communist**  Croatia  Czech Republic  Estonia  Hungary  Lithuania  Latvia  Poland  Russia  Slovenia  Ukraine | | 1  (Zambon et al) |
| Chung and Muntaner (2007) | 19 countries  Based on Huber and Stephens (2001)   - Based on political tradition | **Liberal**  Canada  Ireland  UK  USA | **Christian democratic**  Austria  Belgium  France  Germany  Italy  Luxembourg  The Netherlands  Switzerland | **Social democratic**  Denmark  Finland  Norway  Sweden | **Wage-earner**  Australia  New Zealand  Japan |  | | | 1  (Chung and Muntaner) |
| Olsen and Dahl (2007) | 21 countries.  (based on Navarro and Shi (2001 which is based on Huber and Stephens 1998)   - Based on political tradition | **Liberal/ Anglo-Saxon**  Canada  Ireland  Great Britain  USA | **Christian democratic/Continental**  Belgium  France  Germany  Italy  The Netherlands  Switzerland | **Nordic/Social democratic**  Austria  Denmark  Finland  Norway  Sweden |  | **Fascist/**  **Southern**  Greece  Portugal  Spain | **East**  Czech Republic  Hungary  Poland  Slovenia | | 1  (Olsen and Dahl) |
| Rostila (2007) | 20 countries  Based on Esping-Andersen and Ferrera | **Liberal**  Ireland  UK | **Conservative/ Corporatist**  Austria  Belgium  France  Germany  Luxembourg  The Netherlands | **Social Democratic**  Denmark  Finland  Norway  Sweden |  | **Mediterranean**  Greece  Italy  Portugal  Spain | **Post-socialist**  Czech Republic  Hungary  Poland  Slovenia | | 1  (Rostila 2007) |
| Eikemo et al (2008) | 21 countries  Based on Ferrera | **Anglo-Saxon**  Ireland  UK | **Bismarckian**  Austria  Belgium  France  Germany  Luxembourg  The Netherlands  Switzerland | **Scandinavian**  Denmark  Finland  Norway  Sweden |  | **Southern**  Italy  Greece  Portugal  Spain | **Eastern European**  Czech Republic  Hungary  Poland  Slovakia | | 6  (Eikemo et al, Bambra and Eikemo, Eikemo et al, Eikemo et al, van der Wel et al, Richter et al) |
| Espelt et al (2008) | 9 countries  Based on Huber et al, further elaborated by Navarro et al ([2004](#_ENREF_52)) et al and Bambra ([2007b](#_ENREF_5)).   - Based on political tradition |  | **Christian democratic** France  Germany  Italy  The Netherlands | **Social democratic**  Austria  Denmark  Sweden |  | **Late democrats**  Greece  Spain |  | | 1  (Espelt 2008) |
| Huijts et al (2010) | (typology corresponds to Espelt and Eikemo according to authors, but I don’t see it) | **Liberal**  Ireland  Israel  UK | **Christian Conservative**  Austria  Belgium  France  Germany  Iceland  Italy  Luxembourg  The Netherlands Switzerland | **Social Democratic**  Denmark  Finland  Norway  Sweden |  | **Former Mediterranean dictatorships**  Cyprus  Greece  Portugal  Spain | **Eastern Europe**  Bulgaria  Czech Republic Hungary Poland Slovenia  Slovakia | **Former Soviet republics**  Estonia  Latvia  Russian Federation Ukraine | 1  (Huijts et al) |
| Karim et al  (2010) | 30 countries  Based on Ferrera and Eikemo et al. | **Anglo-Saxon**  Australia  Canada  Ireland  New Zealand  UK  USA | **Bismarckian**  Austria  Belgium  France  Germany  Luxembourg  The Netherlands  Switzerland | **Scandinavian**  Denmark  Finland  Norway  Sweden |  | **Southern**  Italy  Greece  Portugal  Spain | **Eastern European**  Czech  Republic Hungary Poland  Slovakia | **East Asia**  Hong Kong  Japan  Republic of Korea  Singapore  Taiwan | 2  (Karim et al, Chuang et al) |
| Popham (2013) | 37 countries  Based on Ferrera (1996) | **Anglo-Saxon**  Australia  England and Wales  Ireland  Israel  Northern Ireland  Scotland  US | **Bismarckian**  Austria  Belgium  Germany  Luxembourg  The Netherlands  Switzerland | **Nordic**  Denmark  Finland  Iceland  Norway  Sweden |  | **Southern European**  Italy  Portugal  Spain | **Eastern European**  Bulgaria  Czech  Republic Hungary Poland  Slovakia  Slovenia | **Ex-Soviet**  Belarus  Estonia  Latvia  Lithuania  Russia  Ukraine **Confucian**  Japan  Taiwan | 1  (Popham) |
