# Supplementary material for: Understanding the role of welfare state characteristics for health and inequalities – an analytical review
Source: BMC Public Health. 2013 Dec 27;13:1234. doi: 10.1186/1471-2458-13-1234 (PMC3909317; doi:10.1186/1471-2458-13-1234)
Supplement: Additional file 4 — Tables of studies used in the review sorted by welfare regime typology. The tables in this file illustrate the data used, the number of countries looked at, health outcome/s, measure of health inequality, typology and main results for each of the main groups; Esping-Andersen, Ferrera, Huber and colleagues, Korpi and Palme and Regional comparisons. [file 1471-2458-13-1234-S4.docx]

**Additional file 4. Tables of studies used in the review sorted by welfare regime typology.**

.. Study measures inequality

Nordic countries win **pop health**

Nordic: smallest **inequalities**

Other countries win **pop health**

Other countries: smallest **inequalities**

No/conflicting conclusions can be drawn about **pop health**

No conclusions can be drawn about **inequalities**

No or little difference **pop health/ different at different tp**

No or little difference i**nequalities**/different at different tp

**Esping-Andersen**

| Bambra (2006).  _______________ | “Health Status and the Worlds of Welfare.” | OECD  1980, 1998 (decommodification data) 2003 (infant mortality data). | 18 | Infant mortality rate. | n/a | Esping-Andersen   - Social democratic - Conservative - Liberal. | Health status varies across welfare states. Infant mortality rates are lower in Social democratic countries compared to Conservative and Liberal.  There is a significant negative relationship between health and labour market decommodification. |
| --- | --- | --- | --- | --- | --- | --- | --- |
| Deeming and Hayes (2012).  _______________ | “Worlds of Welfare Capitalism and Wellbeing: A Multilevel Analysis.” | World Values Survey.  2000-2005. | 18(?) | Self-reported happiness. | n/a | Esping-Andersen and Castles   - Social democratic - Conservative - Liberal; and - Radical | Respondents living in Liberal and Conservative countries experience at least twice the odds of unhappiness of those living in Social democracies. The differences between the regime types are highly statistically significant. |
| Harding et al. (2012).  _______________  _women__men___  _______________ | “Trends in mortality by labour market position around retirement ages in three European countries with different welfare regimes.” | Office for National Statistics Longitudinal Study (ONS-LS), Turin Longitudinal Study (TLS) and the Finnish linked register study (FS).  1970-2006. | 3 | Mortality rate. | Employment status | Esping-Andersen   - Finland (SD) - Turin (C) - England and Wales (L). | The overall mortality rate was lowest in Turin at all four time points for men. For women it was lowest in Finland.  The mortality rate of those who are not in employment was higher in all three regimes. The mortality rate for those who are not in employment was lowest in Turin and highest in Finland. |
| Kangas (2010).  _______________ | "One hundred years of money, welfare and death: mortality, economic growth and the development of the welfare state in 17 OECD countries 1900-2000." | Human Mortality Database; Mitchell; The World Economy: Historical Statistics; Tanzi and Schuknecht, Lindert; ILO; OECD; SCIP  1900-2000. | 17 | Life expectancy at birth.  Change in life expectancy at birth. | n/a | Esping-Andersen   - Social democratic - Conservative - Liberal | The Nordic group has had a clear lead in longevity for women and men up to the 1960s, after which the other regime types have been able to close the gap and Central Europe seems to surpass the Nordic records. |
| Raphael (2012).  _______________  _______________ | “The political economy of health promotion: part 2, national provision of the prerequisites of health.” | OECD data. | 21 | Infant mortality.  Life expectancy.  (homicide and suicide rates). | n/a | Esping-Andersen (not specified but has similarities with EA and Ferrera)   - Social democratic - Liberal - Conservative - Latin welfare states | Social democratic countries generally have the lowest infant mortality rates and Liberal states the highest.  No obvious pattern is seen between average life expectancy and welfare state type. |
| Rostila (2007).  _______________ | "Social capital and health in European welfare regimes: a multilevel approach." | ESS.  2002 and 2003. | 20 | Self-rated health.  Life expectancy at birth. | n/a | Rostila (based on Esping-Andersen, Ferrera and addition of post socialist regime)   - Social Democratic - Liberal - Conservative-Corporatist - Mediterranean - Post-socialist | Poor perceived health was observed in post-socialist regimes, and also to some extent in Mediterranean regimes. The Liberal regimes had the highest level of self-rated health, followed by Social democratic countries.  Life expectancy is lowest in Post-socialist countries, followed by the Liberal regime. It is highest in the Mediterranean countries. |
| Sekine et al. (2009).  _______________  _______________  _______________ | "Socioeconomic inequalities in physical and mental functioning of British, Finnish, and Japanese civil servants: role of job demand, control, and work hours." | The Whitehall II study, the JACS-study, the Helsinki Health Survey.  1991-1993, 2000-2001, 2003. | 3 | The Short-Form 36:   - Physical health - Mental health. | SES:   - Employment grade | Esping-Andersen   - Social democratic - Liberal - Conservative | Mean physical functioning seems to be lowest in Britain and mean mental functioning seems to be lowest in Finland. Japan receives the highest scores for both health measures.  The lower the SES, the poorer the physical functioning in all cohorts for men and women. The grade differences among non-manual workers were somewhat smaller in the Finnish cohort compared to the British and the Japanese cohorts.  Inequalities regarding poor mental functioning differed among the 3 cohorts. In the British and Japanese cohorts, low grade male employees were more likely to have poor mental functioning. For women, no consistent differences were observed. In the Finnish cohort, reverse associations were found in both men and women (the lower SES, the better the health). |
| Sacker et al. (2011).  _______________  _______________  _______________ | "Social influences on trajectories of self-rated health: evidence from Britain, Germany, Denmark and the USA." | US Panel Study of Income Dynamics; British Household Panel Survey; German Socio-Economic Panel Survey; European Community Household Panel Survey.  1995-2001. | 4 | Self-rated health. | Social position | Castles and Mitchell   - Social democratic - Conservative - Liberal - Radical | Inequalities by social position in self-rated health at 25 (baseline) were larger in Radical and Liberal states compared to social democratic states. Surprisingly, inequalities by social position in self-rated health over time were larger in social democratic countries compared to Conservative but still less than Radical and Liberal. |
| Zambon et al. (2006).  _______________  _______________ | "Do welfare regimes mediate the effect of socioeconomic position on health in adolescence? A Cross-national comparison in Europe, North America, and Israel." | WHO Health Behaviour in School-Age Children Survey.  2001-2002. | 31 | - Self-rated health - General well-being - Health behaviours - Health symptom (Sx) load. | SEP (relative):   - FAS (Family affluence scale) | Zambon et al (based on EA)   - Social Democratic - Liberal - Conservative - Mediterranean/Southern - Eastern/post-Communist | Inequalities in self-rated health are smaller in Social democratic countries compared to Conservative and Southern but not smaller than in Liberal and Eastern regimes.  Inequalities in wellbeing and health symptom load are smaller in Social democratic countries compared to Conservative but not smaller than in Liberal and Southern regimes. |

**Ferrera**

| Bambra et al. (2009).  ……………………….  ????????????????  _______________ | "Gender, health inequalities and welfare state regimes: a cross-national study of 13 European countries." | EUROTHINE.  1998-2004. | 13 | Self-rated health. | Gender.  Education. | Ferrera et al   - Scandinavian - Bismarckian - Anglo-Saxon - Southern European | Women in the Social Democratic and Southern welfare states were more likely to report worse self-rated health than men. In some countries (Italy, Portugal, Sweden), poor self-rated health tended to be more prominent for women with the highest level of education.  No gender differences were found in the Corporatist countries.  There was no consistent welfare state regime patterning for gender differences in self-rated health by education. Variation by country rather than welfare regime. |
| --- | --- | --- | --- | --- | --- | --- | --- |
| Bambra et al. (2010).  _______________  _______________  _______________ | "Welfare state regime life courses: the development of western European welfare state regimes and age-related patterns of educational inequalities in self-reported health." | ESS.  10-year birth cohorts from 1930s to 1980s.  2002, 2004, 2006. | 17 | Self-rated health.  Limiting longstanding illness. | Education | Ferrera et al   - Scandinavian - Bismarckian - Anglo-Saxon - Southern European | Inequalities in longstanding limiting illness were larger in Southern regimes compared to Social democratic (1930s cohort women, 1940s cohort men).  Inequalities in self-rated health were larger in Social democratic regimes compared to Bismarckian (1940s cohort women), Anglo-Saxon (1950s cohort, women) and Southern (1980s cohort, women). Inequalities in limiting longstanding illness were also larger in Social democratic countries compared to Anglo-Saxon (1960s cohort, women). |
| Bambra and Eikemo (2009).  _______________  _______________ | "Welfare state regimes, unemployment and health: a comparative study of the relationship between unemployment and self-reported health in 23 European countries." | ESS.  2002, 2004. | 23 | Self-rated health.  Limiting long-standing illness. | Employment status (relative) | Eikemo et al (based on Ferrera)   - Scandinavian - Bismarckian - Anglo-Saxon - Southern - Eastern European | In all countries, unemployed people reported higher rates of poor health than those in employment. There were also clear differences by welfare state regime: relative inequalities were largest in Anglo-Saxon, Bismarckian and Scandinavian regimes.  For women, inequalities in self-rated health were larger in Scandinavian countries compared to Bismarckian, Southern and Eastern. Inequalities in limiting longstanding illness were also larger for women in Scandinavian countries compared to Southern. |
| Chuang et al. (2012).  _______________  _______________ | “Welfare state regimes, infant mortality and life expectancy: integrating evidence from East Asia.” | OECD Data, World Development Indicators and Asian Development Bank’s key indicators.  1980-2006. | 31 | Infant mortality.  Life expectancy. | n/a | Karim et al (based on Ferrera)   - Scandinavian - Anglo-Saxon - Bismarckian - Southern - Eastern European - East Asian | Scandinavian welfare states have the lowest infant mortality rates, followed by East Asian states, and Eastern European have the highest.  East Asian welfare states have the highest life expectancy followed by Bismarckian and Southern European states. Eastern European welfare states have the lowest. |
| Dragano et al. (2010).  _______________ | “Welfare regimes, labour policies and unhealthy psychosocial working conditions: a comparative study with 9917 older employees from 12 European countries.” | SHARE and the English Longitudinal Study on Ageing.  2004 and 2006. | 12 | Psychosocial quality of work.  Depressive symptoms. | n/a | Ferrera et al   - Scandinavian - Conservative - Liberal - Southern European | Between-country variations in psychosocial quality of work are largely explained by macro indicators and welfare regimes, with poorer psychosocial quality of work in countries with less emphasis on older workers’ protection.  In Liberal and Southern welfare regimes, quality of work matters more for depressive symptoms.  Active labour policies and reliable social protection measures (e.g., Scandinavian welfare regime) exert beneficial effects on the health and well-being of older workers. |
| Eikemo et al. (2008).  _______________  _______________  _______________ | "Welfare state regimes and income-related health inequalities: a comparison of 23 European countries." | ESS.  2002 and 2004. | 23 | Self-rated health.  Limiting long-standing illness. | Income | Eikemo et al (based on Ferrera et al)   - Scandinavian - Anglo-Saxon - Bismarckian - Southern - Eastern European | Limiting longstanding illness was lowest in the Southern regime. The best general health was found in the Anglo-Saxon welfare regime. The Scandinavian regime seems to hold an average position. The poorest health was found in Eastern Europe.  The magnitudes of income-related health inequalities vary by welfare state regime. Inequalities in limiting longstanding illness and self-rated health were smallest in Bismarckian countries, followed by Scandinavian countries (for men). Inequalities were largest in Anglo-Saxon regimes. |
| Eikemo et al. (2008).  _______________  _______________  _______________ | "Health inequalities according to educational level in different welfare regimes: a comparison of 23 European countries." | ESS.  2002-2004. | 23 | Self-rated health.  Limiting long-standing illness. | Education (absolute, relative) | Eikemo et al (based on Ferrera et al)   - Scandinavian - Anglo-Saxon - Bismarckian - Southern - Eastern European | Eastern welfare regimes have the most limiting longstanding illness and poor self-rated health. The southern regimes report the lowest amount of limiting long-standing illness and the Bismarckian regime report the best self-rated health.  Inequalities in limiting longstanding illness were larger in Southern regimes compared to Scandinavian (for women) and inequalities in Bismarckian regimes were even smaller.  Inequalities in self-rated health were larger in Scandinavian countries compared to Bismarckian (both men and women) and Anglo-Saxon (for women). |
| Eikemo et al. (2008).  _______________ | "Welfare state regimes and differences in self-perceived health in Europe: a multilevel analysis." | ESS.  2002-2004. | 21 | Self-rated health. | n/a | Eikemo et al (based on Ferrera et al)   - Scandinavian - Anglo-Saxon - Bismarckian - Southern - Eastern European | Anglo-Saxon welfare regimes have the best self-perceived general health followed by the Scandinavian regimes in comparison to Southern and East European welfare regimes. Bismarckian regimes hold an average position. |
| Huijts et al. (2010).  _______________ | “Political Regimes, Political Ideology, and Self-Rated  Health in Europe: A Multilevel Analysis.” | ESS.  2002,2004,2006. | 30 | Self-rated health. | n/a | Huijts et al (typology corresponds to Espelt and Eikemo acc to authors…??)   - Social Democratic - Christian Conservative - Liberal - Former Mediterranean dictatorships - Eastern Europe - Former Soviet republics | Individual ideology and political regime are independently associated with self-rated health. Individuals with right-wing ideologies report better health than left-wing individuals.  Respondents from Eastern Europe and former Soviet republics report poorer health than individuals from Social democratic, Liberal, Christian conservative, and former Mediterranean dictatorship countries. Levels of self-rated health are similar in these countries, but Social democratic countries do not have the best self-rated health as is expected. |
| Karim et al. (2010).  _______________  _______________ | "Welfare state regimes and population health: Integrating the East Asian welfare states." | World Factbook; OECD data, Asian development Bank, Statistical Yearbook of the Republic of China, WHO data, Nations Master, Australian Institute of Health and Welfare Health Expenditure database.  2003. | 30 | Infant mortality.  Life expectancy at birth. | n/a | Karim et al (based on Ferrera et al.)   - Scandinavian - Anglo-Saxon - Bismarckian - Southern - Eastern European - East Asian | The Scandinavian welfare state regime had the lowest average infant mortality rate and the Eastern European had the highest. The east Asian regime had the highest average life expectancy whilst the Eastern European had the lowest average. |
| Popham et al. (2013).  ……………………_.  _men___women_  _young___old___  _men___women_ | “Are health inequalities really not the smallest in the Nordic welfare states? A comparison of mortality inequality in 37 countries.” | Human Mortality Database.  2006 | 37 | Life expectancy…  Mortality  Life expectancy | Total inequality= average life lost per death | Popham et al (based on Ferrera et al.)   - Nordic - Bismarckian - Anglo-Saxon - Southern European - Eastern European - Ex-Soviet - Confucian | Life expectancy is the highest in the Nordic countries for men, and for women it is highest in the Confucian countries.  Nordic countries had lower younger age mortality but not older age mortality.  Inequalities in life expectancy in the Nordic countries are smallest for men, and for women inequalities they are smallest in southern Europe. |
| Richter et al. (2012).  _______________  _______________  _______________  _______________ | “Welfare state regimes, health and health inequalities in adolescence: a multilevel study in 32 countries.” | WHO Health Behaviour in School-aged Children.  2006. | 32 | Self-rated health.  Health complaints. | SEP:   - Family affluence - Parental occupation | Eikemo et al (based on Ferrera)   - Scandinavian - Anglo-Saxon - Bismarckian - Southern - Eastern European | The Eastern and Anglo-Saxon regimes had the highest prevalence for fair⁄poor self-rated health and the lowest rates were found in the Southern regime. The prevalence of health complaints gave a different picture: adolescents in the Southern and Eastern regimes reported the higher prevalence compared to the Scandinavian regime who showed the lowest rate.  No results concerning higher odds ratios for either health outcomes was found for adolescents belonging to the different SEP groups in the Anglo-Saxon and Eastern regimes compared to the corresponding SEP groups in the Scandinavian regime. |
| Van der Wel et al. (2012).  _______________  _______________  _women___men _ | “Social inequalities in "sickness": does welfare state regime type make a difference? A multilevel analysis of men and women in 26 European countries.” | EU-SILC. | 26 | Limiting long-standing illness. | Education | Eikemo et al (based on Ferrera)   - Scandinavian - Anglo-Saxon - Bismarckian - Southern - Eastern European | The prevalence of longstanding limiting illness is lowest in Scandinavian regimes and highest in Anglo-Saxon regimes.  The probability of non-employment, for men and women reporting limiting longstanding illness in combination with low educational level, was particularly high in the Anglo-Saxon and Eastern welfare regimes, and lowest in the Scandinavian regime.  For men, absolute and relative social inequalities in sickness were lowest in the Southern regime and for women inequalities were lowest in Scandinavian regimes. |

**Huber and colleagues**

| Borrell et al. (2009).  _______________  _men___women__ | "Analyzing differences in the magnitude of socioeconomic inequalities in self-perceived health by countries of different political tradition in Europe." | OECD data, EUROTHINE and LIS.  2000. | 13 | Self-rated health. | Education | Navarro, Schmitt, and Astudillo (based on Huber et al)   - Social Democratic - Christian Democratic - Liberal - Late Democracy | Educational-level inequalities in health exist in all countries and in all political traditions.  Inequalities in health were smaller in Social democratic countries compared to Christian democratic, Liberal and Late democracies, for men. For women, inequalities were larger in Social democratic countries compared to Late democracies. |
| --- | --- | --- | --- | --- | --- | --- | --- |
| Chung and Muntaner (2007).  _______________ | "Welfare state matters: a typological multilevel analysis of wealthy countries." | OECD data, UN Common Statistical Database.  1960-1994. | 19 | Infant mortality.  Low birth weight. | n/a | Chung and Muntaner (based on Huber and Stephens)   - Social Democratic - Christian Democratic - Liberal - Wage-earner | Infant mortality rate is lower in Social democratic countries compared to other regimes. Low birth weight prevalence is more common in Christian democratic, Liberal and Wage-earner regimes compared to Social democratic. |
| Espelt et al. (2008).  _______________  _______________  _______________  _______________ | "Inequalities in health by social class dimensions in European countries of different political traditions." | SHARE.  2004. | 9 | Self-rated health.  Limiting longstanding illness. | Social class:   - Ownership - Education - Management | Espelt et al (Based on Huber et al, further elaborated by Navarro (2004) et al. and Bambra (2007)   - Social Democratic - Christian Democratic - Late Democratic | Self-rated health was the best in Social democratic regimes and lowest in Late democracies (men and women) and Christian democracies (women). There was little difference in long-term illness between the welfare regimes.  Health inequalities by social class are found in all three political traditions. Differences in all three dimensions of social class are more marked in Late democracies and mainly among women.  Inequalities in education for both self-rated health and limiting longstanding illness were higher in Social Democratic countries compared to Christian democratic (for men). |
| Navarro et al. (2006).  ……………………….  _______________ | "Politics and health outcomes." | OECD data; ILO-LABORSTA; Müller and Strom; Alcantara Saez; Caramani; Comparative Welfare State dataset; WHO data.  1972-1996. | 17 | Infant mortality rate.  Life expectancy at birth. | Income distribution (Theil index) | Navarro (based on Huber and Stephens)   - Social Democratic - Christian Democratic - Late Democratic - Former dictatorship | Political parties with egalitarian (Social democratic) ideologies tend to implement redistributive policies, which reduce social inequalities and are important in reducing infant mortality and, to a lesser degree, in increasing life expectancy. |
| Olsen and Dahl (2007).  _______________ | ”Health differences between European countries.” | ESS.  2003. | 21 | Self-rated health. | n/a | Olsen and Dahl (based on Navarro and Shi which is based on Huber and Stephens)   - Anglo-Saxon - Nordic - Continental - South - East | The East European countries have the lowest levels of health. There are no significant differences between the other groups of European countries, when categorised into regime types. |

**Korpi and Palme**

| Sanders et al. (2009).  _______________  _______________  _______________ | "A cross-national comparison of income gradients in oral health quality of life in four welfare states: application of the Korpi and Palme typology." | Adult Dental Survey (UK); Health Examination Survey (Finland); National Dental telephone Interview Survey (Germany and Australia).  1998-2002. | 4 | Oral health impact profile (OHIP-14) severity scores. | Household income | Korpi and Palme   - Targeted - Corporatist - Basic Security (L) - Encompassing (SD) - Voluntary State Subsidised | Average dental health scores were better in Finland, followed by Germany and the UK, and were highest in Australia.  Inequalities in oral health were larger in the Encompassing regime type (Social democratic) compared to the Corporatist. Significant income gradients in oral health were found in all countries except Germany. |
| --- | --- | --- | --- | --- | --- | --- | --- |

**Geographical comparisons**

| Avendano et al. (2009).  ..  _______________ | "Educational level and changes in health across Europe: longitudinal results from SHARE." | SHARE.  2004/2005, 2006/2007. | 10 | Self-rated health.  Limiting longstanding illness.  ≥1 chronic disease.  Depression.  ≥1 activity limitation. | Education. | 3 groups that roughly represent different welfare state regimes, geographical regions and behavioural patterns:   - Northern - Southern - Western Europe | There is some evidence that educational effects on health are significant for most outcomes in western and southern Europe, and that they are weaker and non-significant in the Nordic countries. |
| --- | --- | --- | --- | --- | --- | --- | --- |
| Granados (2010).  _______________ | "Politics and health in eight European countries: a comparative study of mortality decline under social democracies and right-wing governments." | WHO Regional Office for Europe; Angus Maddison’s databank; Statistics Iceland; OECD data; UN; University of California at Berkeley and Max Planck Institute for Demographic research; International Smoking Statistics Database.  1950-2000. | 8 | Infant mortality rate.  Life expectancy at birth.  Age-specific death rate. | n/a | Nordic and Southern European. | By the early 21^st^ century Greece, Portugal and Spain have arrived at basically the same levels of population health as the Nordic countries, despite starting off a very different levels. The Nordic countries are however, still in the lead regarding all 3 health measures. |
| Hoffmann (2011).  ..  _______________ | “Socioeconomic inequalities in old-age mortality: A comparison of Denmark and the USA.” | Danish register data and the US Health and Retirement Study.  1980-2002 (DK) and 1992-2006 (US). | 2 | Mortality. | SES   - education - income | Denmark (SD) and USA (L). | Socioeconomic differences in mortality were larger in Denmark than in USA even after controlling for a number of covariates. Low income seems to be a more powerful risk factor for mortality than low education. |
| Kunst et al. (2005).  ..  _______________  _______________ | "Trends in socioeconomic inequalities in self-assessed health in 10 European countries." | Nationally representative health interview surveys.  1980s and 1990s. | 10 | Self-rated health. | Social class   - - Education - - Income (absolute, relative). | 10 different European countries. | The highest rates of poor self-rated health are found in Finland and Italy. The best health is, in general, found in Sweden, Norway and Denmark.  Socioeconomic inequalities in self-rated health show a high degree of stability in European countries between the 1980’s and 1990’s. Education-based absolute and relative health differences (the only indicator available for all Nordic countries) were stable in the Nordic countries. Increased inequalities were observed in Spain, Italy, and The Netherlands.  Income-based absolute health inequalities increased during the same time period and they were most clear for Italian and Spanish men and women, and for Dutch women. |
| Olafsdottir (2007).  ..  _______________  _______________ | "Fundamental causes of health disparities: stratification, the welfare state, and health in the United States and Iceland." | General Social Survey and Health and Living Standards of Adult Icelanders survey.  1998. | 2 | Self-rated health. | SEP (absolute and relative) | Iceland (SD) and USA (L). | Younger people report better health in Iceland than in USA. Self-rated health is similar at age 40 and by age 50, Americans have surpassed the Icelanders.  Health inequality (relative and absolute) exists in the United States and Iceland, but the effects of affluence and health are weaker in Iceland, and having a more vulnerable family structure is less harmful in Iceland than in the United States. |
| Ploubidis et al. (2012).  ..  _______________  _______________ | “Later life health in Europe: how important are country level influences?” | SHARE. | 14 | Self-rated health. | Gini coefficient (income inequality). | - Social democratic (Sweden, Denmark, and the Netherlands) - Others (Austria, Germany, Switzerland, Belgium, Ireland, Spain, Italy, France, Greece, Czechia, Poland) | Social democratic countries exhibit better health status than the other countries and this effect is largely mediated by more equal income distribution. Sweden and Denmark have the lowest Gini coefficient score. A higher score seems to have a negative association with health. |
