# Supplementary material for: Understanding the role of welfare state characteristics for health and inequalities – an analytical review
Source: BMC Public Health. 2013 Dec 27;13:1234. doi: 10.1186/1471-2458-13-1234 (PMC3909317; doi:10.1186/1471-2458-13-1234)
Supplement: Additional file 5 — Tables of studies used in the review sorted by Institutional approach. The tables in this file illustrate the different institutional approaches used in the studies. They are sorted by type of policy (pension benefits, economic assistance and unemployment benefits, family benefits, and access to health care), the data used, health outcome/s, measure of health inequality, and main results for each of the main groups. [file 1471-2458-13-1234-S5.docx]

**Additional file 5. Tables of studies used in the review sorted by institutional approach.**

**Pension benefits**

| Esser and Palme (2010). | “Do public pensions matter for health and wellbeing among retired persons? Basic and income security pensions across 13 Western European countries”. | SCIP (2000 and 2002/03) and ESS (2004/05). | Self-rated health. | n/a | Pension benefits   - basic security - income security | Better health and wellbeing is found in countries with more generous pensions (although effects are only significant for men). For men’s excess ill-health, income security pensions are more important. Women health is more strongly associated with the generosity of basic security pensions. |
| --- | --- | --- | --- | --- | --- | --- |
| Kangas (2010). | "One hundred years of money, welfare and death: mortality, economic growth and the development of the welfare state in 17 OECD countries 1900-2000." | SCIP, OECD, Human Mortality Database, ILO, Mitchell, The World Economy: Historical Statistics, Tanzi and Schuknecht, Lindert,.  1900-2000. | Life expectancy at birth.  Change in life expectancy at birth. | n/a | Pensions | There is an association between increases in basic pensions and higher life expectancy. |
| Lundberg et al. (2008). | "The role of welfare state principles and generosity in social policy programmes for public health: an international comparative study." | SCIP, OECD, Human Mortality database, Angus Maddison’s databank, ILO, WHO mortality database.  1970-2000 and 1950-2000. | Old-age excess mortality rate. | n/a | Basic security pension   - encompassing - state corporatist - basic - targeted | Generosity in basic security type of pensions is linked to lower old-age excess mortality, but not in income security pensions. |
| Norstrom and Palme (2010). | "Public pension institutions and old-age mortality in a comparative perspective." | SCIP, Human Mortality Database, Angus Maddison’s data bank, WHO Mortality Database.  1950-2000. | Old-age excess mortality rate. | n/a | Pension benefits   - basic security - income security | Significant results suggest that basic security (but not income security) has a beneficial impact on old-age excess mortality. |

**Economic assistance and Unemployment benefits**

| Ferrarini and Sjoberg (2010). | “Social policy and health: transition countries in a comparative perspective”. | SCIP, Parental Leave Database, SPEC, LIS, ESS.  Mid 1990s- mid 2000s. | Self-rated health.  Infant mortality. | n/a | Unemployment benefits. | A system of universal unemployment policies, which incorporates a large share of the population and provides a high degree of earnings replacement in case of unemployment, seems beneficial for all, not only for the unemployed, but also for the health of the employed. |
| --- | --- | --- | --- | --- | --- | --- |
| Fritzell et al. (2007). | "From macro to micro: the health of Swedish lone mothers during changing economic and social circumstances." | ULF.  1985,1990,1996 | Self-rated health.  Hospitalisation.  All cause and cause-specific mortality.  Severe morbidity. | Type of mother (lone vs. coupled relative). | Social assistance (cash assistance). | Relative difference between lone and coupled mothers was constant over time, despite welfare-state changes in Sweden.  The authors concluded that the Swedish welfare state perhaps buffers economic pressure, despite cuts. |
| Nordenmark et al. (2006). | "The impact of unemployment benefit system on the mental well-being of the unemployed in Sweden, Ireland and Great Britain." | Long-term Unemployment project (Sweden 1992-1997), Living in Ireland Panel Survey (1994-2001), British Household Panel Survey (1994-2001). | Psychological distress in General Health Questionnaire. | n/a | Type of unemployment benefit   - none - flat rate - income replacement | The type of benefit received is an important determinant of mental distress with income replacement benefits being more beneficial than flat rate benefits.  Systems impact differentially on different groups with income replacement benefits tending to maintain pre-unemployment differences in distress and flat rate benefits equalising these differences. |

| Burstrom et al. (2010). | “Health inequalities between lone and couple mothers and policy under different welfare regimes - The example of Italy, Sweden and Britain." | Swedish survey of Living Conditions (1999-2001), General Household Survey (Britain 2000/2001), National Health Survey (Italy 1999-2000). | Self-rated health.  Limiting Longstanding illness. | Type of mother (lone vs. coupled). | Family policy models   - market-oriented family - dual-earner - contradictory - general-family | Dual-earner family policy model and generous family benefits provide more protection to Swedish mothers in general and lone mothers in particular from poor health, poverty, and unemployment. |
| --- | --- | --- | --- | --- | --- | --- |
| Engster and Olofsdotter Stensota (2011). | “Do Family Policy Regimes Matter for Children's Well-Being?” | OECD, UNICEF, Gauthier and Bortnik’s Comparative Maternity, Parental, and Childcare Database, LIS | Child mortality.  Child poverty.  Educational attainment and achievement. | n/a | Family policies   - Family cash and tax benefits - Paid parenting leaves - Public child care support | Family policies have a significant impact on improving children’s well-being, and dual-earner regimes, combining high levels of support for paid parenting leaves and public child care, are strongly associated with low levels of child poverty and child mortality. |
| Ferrarini and Norstrom (2010). | "Family policy, economic development and infant mortality: a longitudinal comparative analysis." | SCIP, Human Mortality Database, Angus Maddison’s data bank, ILO, OECD, Parental Leave Database,  1950-2000. | Infant mortality rate. | n/a | Family policy benefits   - earning-related parental insurance - child leave benefits - child allowances - maternity grants - tax reductions for a worker with a dependant spouse | The more generous the earnings-related parental leave benefits, the lower the infant mortality (for the period 1970-2000). |
| Ferrarini and Sjoberg (2010). | “Social policy and health: transition countries in a comparative perspective”. | SCIP, Parental Leave Database, SPEC, LIS, ESS.  Mid 1990s- mid 2000s. | Self-rated health.  Infant mortality. | n/a | Family policy benefits   - child allowance - marriage subsidy - parental insurance - childcare leave - maternity grant | Universal family policies seem to be beneficial for all, not only those for those who use them. |
| Lundberg et al. (2008). | "The role of welfare state principles and generosity in social policy programmes for public health: an international comparative study." | SCIP, Human Mortality database, Angus Maddison’s databank, ILO, OECD, WHO mortality database.  1970-2000 and 1950-2000. | Infant mortality rate. | n/a | Family policy models   - dual-earned - general - market oriented | Increased generosity in family policies that support dual-earner families is linked with lower infant mortality rates. |
| Tanaka (2005) | “Parental leave and child health across OECD countries”. | ILO; the US Social Security Administration; WHO; OECD; Work Life Research Centre.  1969-2000. | Infant mortality.  Child mortality.  Low birth weight.  Immunisation. | n/a | Parental leave policies. | The extension of weeks of job-protected paid leave has significant effects on all mortality rates. Other (unpaid) leave does not have a significant effect on improving infant health. |

**Family benefits**

**Access to health care**

| Borrell et al. (2006). | "AIDS mortality before and after the introduction of highly active antiretroviral therapy: does it vary with socioeconomic group in a country with a National Health System?" | 1991-2001 | AIDS mortality. | Education (relative). | Introduction of free HAART. | Education-based relative inequalities before and after the introduction of HAART were stable. This suggests that perhaps access to HAART, or adherence, is lower than desirable, in people of lower SES groups. |
| --- | --- | --- | --- | --- | --- | --- |
| James et al. (2007). | “Avoidable mortality by neighbourhood income in Canada: 25 years after the establishment of universal health insurance." | Canadian Mortality Database and population censuses.  1971, 1986, 1991, 1996. | Mortality (amenable vs. non-amenable causes). | Income (absolute). | Establishment of universal insurance for doctors (1968) and hospital services (1957). | Income-based absolute inequality in mortality amenable to medical care decreased substantially.  Absolute inequality in mortality amenable to public health increased somewhat. |
| Korda et al. (2007). | "Differential impacts of health care in Australia: trend analysis of socioeconomic inequalities in avoidable mortality." | Australian Bureau of Statistics.  1986, 1991, 1996, 2001. | Avoidable and non-avoidable mortality. | Socioeconomic status (absolute, relative). | Australian health care system (Medicare). | Health care has contributed to decreasing the absolute socioeconomic mortality gap. However, advantaged people have obtained a disproportionate benefit of health care, contributing to widening relative health inequalities. |
