# Supplementary material for: Understanding the role of welfare state characteristics for health and inequalities – an analytical review
Source: BMC Public Health. 2013 Dec 27;13:1234. doi: 10.1186/1471-2458-13-1234 (PMC3909317; doi:10.1186/1471-2458-13-1234)
Supplement: Additional file 6 — Tables of studies used in the review sorted by Expenditure approach. The tables in this file illustrate the two Expenditure approaches used in the studies. They are sorted by type of spending (health spending or social spending), the data used, health outcome/s, measure of health inequality, and main results for each of the main groups. [file 1471-2458-13-1234-S6.docx]

**Additional file 6. Tables of studies used in the review sorted by expenditure approach.**

**Social spending**

| Bradley et al. (2011). | “Health and social services expenditures: associations with health outcomes.” | OECD data.  2009 and 1995-2005. | Life expectancy at birth.  Infant mortality.  Low birth weight.  Maternal mortality.  Potential years of life lost. | n/a | Social services expenditures. | Social services expenditures adjusted for GDP were significantly associated with better health outcomes in 3/5 indicators (life expectancy, infant mortality and potential years of life lost).  The ratio of social expenditures to health expenditures was significantly associated with better outcomes in infant mortality, life expectancy and increased potential life years lost, after adjusting for the level of health expenditures and GDP. |
| --- | --- | --- | --- | --- | --- | --- |
| Dahl and van der Wel (2012). | “Educational inequalities in health in European welfare states: A social expenditure approach.” | EU-SILC. | Self-rated health. | Education. | Social spending:  - gross public social expenditure in % of GDP  - net total social expenditure in % of GDP  - gross public social expenditure in purchasing power parities  - net total social expenditure in purchasing power parities | Social expenditures are associated with lower health inequalities among women and, to a lesser degree, among men. Especially those with primary education benefit from high social transfers as compared with those who have tertiary education. This means that lower educational inequalities in health, in absolute and relative terms, are linked to higher social spending. The four different operationalisations of social spending produce similar patterns. |
| Kangas (2010). | "One hundred years of money, welfare and death: mortality, economic growth and the development of the welfare state in 17 OECD countries 1900-2000." | OECD, Human Mortality Database, Mitchell, The World Economy: Historical Statistics, Tanzi and Schuknecht, Lindert, ILO, SCIP.  1900-2000. | Life expectancy at birth.  Change in life expectancy at birth. | n/a | Public social security spending.  Generosity and degree of universalism. | Relationships between social spending and life expectancy vary from cross-section to cross-section. Initial investment in social policy leads to increase in life expectancy but after a certain level of spending, the extra spending does not contribute that much. |
| Stuckler et al. (2010). | “Budget crises, health, and social welfare programmes.” | OECD data.  1980-2005. | Mortality. | n/a. | Social welfare spending. | A rise in social welfare spending was associated with over a sevenfold greater reduction in mortality.  There was no observable protective effect associated with general government spending, which is understandable since military, prison, or similar spending would not be expected to have a visible public health effect. |

**Health spending**

| Bradley et al. (2011). | “Health and social services expenditures: associations with health outcomes.” | OECD data.  2009 and 1995-2005. | Life expectancy.  Infant mortality.  Low birth weight.  Maternal mortality.  Potential life years lost. | n/a | Health expenditures   - % of GDP | Health services expenditures adjusted for GDP per capita were significantly associated with better health outcomes in only 2/5 health indicators (life expectancy and maternal mortality).  The ratio of social expenditures to health expenditures was significantly associated with better outcomes in infant mortality, life expectancy and increased potential life years lost, and worse health outcomes in low birth weight after adjusting for level of health expenditures and GDP. |
| --- | --- | --- | --- | --- | --- | --- |
| Gesthuizen et al. (2012) | “Explaining health marginalisation of the lower educated: the role of cross-national variations in health expenditure and labour market conditions.” | ESS.  2002-2008. | Self-rated health. | Education. | Governmental health expenditure. | The relative risk of lower educated individuals being in poor health is smaller in countries where the government spends much on healthcare and with a highly modernised labour market. |
| Olsen and Dahl (2007). | ”Health differences between European countries.” | ESS.  2003. | Self-rated health. | n/a | Public social spending on health.  Country-level characteristics e.g. GDP. | Public social spending on health is negatively correlated with health for women and unrelated for men. A possible explanation is that because OECD countries already have high levels of expenditure on health, additional spending will have little effect. GDP per capita has a positive curvilinear association with health. |
| Vavken et al. (2012). | “Does increased health care spending afford better health care outcomes?  Evidence from Austrian health care expenditure since the initiation of DRGs.” (a reimbursement system for health care provision) | Statistics Austria, 2009 Manual of the Austrian Social Insurance, OECD data and Eurostat.  1997. | Mortality.  Years of life lost. | n/a. | Health care expenditure   - total - total public - total private - total spent in prevention. | Health care spending was associated with mortality and years of life lost reduction. The strongest association among the independent variables was seen for spending for prevention. The strongest association for the dependent variables was seen for cardiovascular disease followed by injuries. Also, socio-economic status was shown to be an important confounder in all studied associations. |
| Wu and Chiang (2007). | “Comparing Child Mortality in Taiwan and Selected Industrialized Countries.” | UNICEF, UN and OECD data.  2002. | Infant mortality rate.  Under 5 mortality rate. | n/a | National health expenditure. | Total health expenditure didn’t explain the cross-national variation in IMR and U5MR. However, a larger public share of health spending was correlated with lower IMR. In industrialised countries where health expenditure primarily comes from public sources, there is generally universal access to health care. Hence, whether or not health care matters to the health of the child population seems to depend on the universal accessibility of health care, indicating the importance of social welfare policies. |
